# Marine endophytic fungi associated with *Halopteris scoparia* (Linnaeus) Sauvageau as producers of bioactive secondary metabolites with potential dermocosmetic application

**DOI:** 10.1371/journal.pone.0250954

**Published:** 2021-05-13

**Authors:** Maria da Luz Calado, Joana Silva, Celso Alves, Patrícia Susano, Débora Santos, Joana Alves, Alice Martins, Helena Gaspar, Rui Pedrosa, Maria Jorge Campos

**Affiliations:** 1 MARE–Marine and Environmental Sciences Centre, Polytechnic of Leiria, Peniche, Portugal; 2 BioISI–Biosystems and Integrative Sciences Institute, Faculty of Sciences of the University of Lisbon, Lisbon, Portugal; 3 MARE–Marine and Environmental Sciences Centre, ESTM, Polytechnic of Leiria, Peniche, Portugal; Institute for Biological Research, SERBIA

## Abstract

Marine fungi and, particularly, endophytic species have been recognised as one of the most prolific sources of structurally new and diverse bioactive secondary metabolites with multiple biotechnological applications. Despite the increasing number of bioprospecting studies, very few have already evaluated the cosmeceutical potential of marine fungal compounds. Thus, this study focused on a frequent seaweed in the Portuguese coast, *Halopteris scoparia*, to identify the endophytic marine fungi associated with this host, and assess their ability to biosynthesise secondary metabolites with antioxidative, enzymatic inhibitory (hyaluronidase, collagenase, elastase and tyrosinase), anti-inflammatory, photoprotective, and antimicrobial (*Cutibacterium acnes*, *Staphylococcus epidermidis* and *Malassezia furfur*) activities. The results revealed eight fungal taxa included in the Ascomycota, and in the most representative taxonomic classes in marine ecosystems (*Eurotiomycetes*, *Sordariomycetes* and *Dothideomycetes*). These fungi were reported for the first time in Portugal and in association with *H*. *scoparia*, as far as it is known. The screening analyses showed that most of these endophytic fungi were producers of compounds with relevant biological activities, though those biosynthesised by *Penicillium* sect. *Exilicaulis* and *Aspergillus chevalieri* proved to be the most promising ones for being further exploited by dermocosmetic industry. The chemical analysis of the crude extract from an isolate of *A*. *chevalieri* revealed the presence of two bioactive compounds, echinulin and neoechinulin A, which might explain the high antioxidant and UV photoprotective capacities exhibited by the extract. These noteworthy results emphasised the importance of screening the secondary metabolites produced by these marine endophytic fungal strains for other potential bioactivities, and the relevance of investing more efforts in understanding the ecology of halo/osmotolerant fungi.

## Introduction

The incessant search for new, safer, eco-friendly and sustainable natural products as alternatives to chemically synthetized products has been revealing a remarkable diversity of bioactive secondary metabolites produced by marine fungi with multiple potential applications in several biotechnological fields. Thus far, more than 3500 marine fungal secondary metabolites were described [1, 2] and a likely higher number remain untapped, considering the marine ecological niches that were not yet explored and the high number of biosynthetic clusters integrated in known fungal species genomes that are silent or cryptic [3–5]. In addition to the novelty, diversity and/or potency of marine bioactive secondary metabolites demonstrated in several studies [1, 5, 6], some of these are also easy to obtain in large-scale laboratory culture conditions, in high yields and reduced time periods [7].

Cosmetic industry represents an example where increasing efforts have been done in the last few years to obtain new formulations with bioactive natural compounds, particularly for cosmeceutical products that have esthetic and dermatological treatment purposes [8].

Regarding specifically anti-ageing products, there is a main concern in including different bioactive ingredients with distinct targets in the general action spectrum in order to maximize the efficiency and beneficial effects of the products in preventing, decelerating or reversing ageing process.

Skin-ageing is a complex process induced and regulated by intrinsic and external factors, that implies deterioration of cutaneous barrier and progressive structural and functional alterations in the skin tissue [9–14]. Intrinsic or cellular ageing is a natural, physiological and time-related process, which might be triggered by several factors, such as telomere shortening, hormonal changes and oxidative stress arising from normal cell metabolism [14]. Intrinsic skin-ageing process involves a gradual: 1) decrease of proliferative capacity of epidermal and dermal cells (keratinocytes, melanocytes and fibroblasts); 2) decrease of biosynthesis of the components of extracellular matrix in dermis responsible for tensile strength, moisture and elasticity of the skin, i.e. protein fibers collagen and elastin, and glycosaminoglycan hyaluronic acid; 3) increase of enzymatic degradation of these components by collagenase, elastase and hyaluronidases. All of these processes contribute to an atrophy of epidermal and dermal layers and formation of fine wrinkles. External ageing is caused by environmental factors, particularly prolonged and intense exposure to ultraviolet (UV) radiation of sunlight. UV-A and UV-B, which represent 5% and 95% of total UV radiation that reaches the Earth´s surface respectively, might stimulate the generation of reactive oxygen species (ROS) that induce an oxidative stress response in cells. High levels of ROS will then activate the molecular cascades responsible for the decrease of collagen synthesis and increase of enzymatic degradation of extracellular matrix proteins, but also will cause damages in DNA structure [14]. Moreover, UV radiation and particularly UV-B might directly cause photochemical modifications in DNA and proteins [15]. Thus, excessive UV radiation has profound negative effects on the skin, being the main responsible for the development of deep lines and coarse wrinkles, rough-textured appearance, pigmentary changes and inflammations on the skin, and skin cancer. The slowing down of intrinsic skin-ageing and prevention of photo-ageing may be obtained through the use of cosmeceutical products that aggregate inhibitors of collagenase, elastase and hyaluronidases, antioxidant compounds and UV-blockers or UV-absorbers [10, 14, 15]. Additionally to these biological activities, the incorporation of compounds with the ability to inhibit the tyrosinase enzyme activity and, consequently, the synthesis and accumulation of melanin and darkening of the skin tone will represent a highly valuable plus in anti-ageing or other cosmeceutical products [16, 17]. On the other hand, bioactive compounds that stimulate tyrosinase activity and the natural production of melanin, as well as compounds that act as UV-filters, could also be relevant for other cosmetic/cosmeceutical formulations, such as sunscreen products with or without potential tanning agents [18].

Another major purpose of the dermocosmetic industry is to find effective solutions to several skin disorders that are triggered by main resident commensal species of the skin microbiome, particularly *Cutibacterium acnes*, *Staphylococcus epidermidis* and *Malassezia* species, when exposed to certain exogenous or endogenous factors. In fact, and even though *C*. *acnes* and *S*. *epidermidis* assume an important role in protecting the healthy skin from pathogens, these bacteria are also involved in the physiopathology of acne. Concretely, the increase of sebum associated to hormonal modifications that occur mostly during puberty, can promote an over-colonization of pilosebaceous unit and skin surface by *C*. *acnes* and a decrease of *S*. *epidermidis*. This disturbance in the skin-microbiota balance may lead to different levels of response by the immune system and different severity levels of inflammatory acne [19]. The intensification of sebaceous gland activity might also induce different *Malassezia* species to cause unique or similar pathologies, such as pityriasis versicolor, *Malassezia* folliculitis, seborrheic dermatitis/dandruff, atopic dermatitis, and psoriasis [20, 21]. Effective cosmeceutical treatments for these skin disorders should favor the use of natural compounds that may contribute to the homeostasis of skin and/or present strong anti-inflammatory and antimicrobial activities against these two bacteria and fungi.

Even though the increasing number of reports of bioactive compounds produced by marine fungi with photoprotective, antioxidative, anti-inflammatory and antimicrobial activities that may potentially be incorporated in the above-referred cosmeceutical formulations, very few secondary metabolites have already been tested with this purpose [7, 17]. Concretely, some bioprospecting studies that focused on marine fungal strains reported *i)* the photoprotective potential of some compounds to be incorporated in sunscreen products, acting either as UV-absorbers or UV-induced free radicals scavengers [7, 17, 22–24], *ii)* the ability of other compounds to inhibit tyrosinase activity and be potentially used in skin-whitening products, such as cyclopentenones myrothenones A and B, kojic acid and other α-pyrone derivatives, eudesmane sesquiterpenes, and homothallin-II [25–28], and *iii)* the antibacterial activity a few different compounds against *C*. *acnes* and *S*. *epidermidis* [29, 30].

The majority of the bioactive secondary metabolites described so far were isolated from generalist halo/osmotolerant fungi occurring within seaweeds and sessile marine invertebrates, that are more exposed to multiple environmental adversities, particularly UV radiation, herbivory, pathogens, daily emersion/immersion fluctuations and/or other factors [1, 31]. In fact, and even though the relationship between these fungi and their hosts is not yet totally understood, apparently ranging from latent pathogenesis to mutualistic symbiosis, it seems to be beneficial for both of them [31–33]. In line with this assumption, the high diversity of bioactive chemical compounds and, particularly, those with antimicrobial and/or antioxidant activities has been attributed to an adaptation strategy of marine fungi to protect their hosts from biotic and abiotic stresses and protect themselves from ROS and free-radicals produced by the host defense-system [16, 31, 34–40].

Thus, this study intended to evaluate the cosmeceutical potential of secondary metabolites biosynthesised by marine endophytic fungi associated with a seaweed, given the lack of studies performed with this purpose, the high number of seaweeds whose fungal communities remain to be inventoried, and the high fungal richness and/or secondary metabolites chemical diversity frequently reported from red and brown seaweeds [35, 41].

Specifically, this study focused on the marine endophytic fungi associated with *Halopteris scoparia* (Linnaeus) Sauvageau, a common and ecologically and biotechnologically relevant seaweed species of the Portuguese coast [42, 43], and their ability to produce secondary metabolites with antioxidative, enzymatic inhibitory (hyaluronidase, collagenase, elastase and tyrosinase), anti-inflammatory, photoprotective, and antimicrobial (*C*. *acnes*, *S*. *epidermidis* and *M*. *furfur*) activities. The cytotoxicity of bioactive crude extracts was also tested to ensure that the compounds were safe to incorporate into cosmeceutical formulations. Additionally, a chemical characterization of the most promising bioactive crude extract was also performed.

## Material and methods

### Collection of seaweeds and isolation of fungi

**Fig 1:**
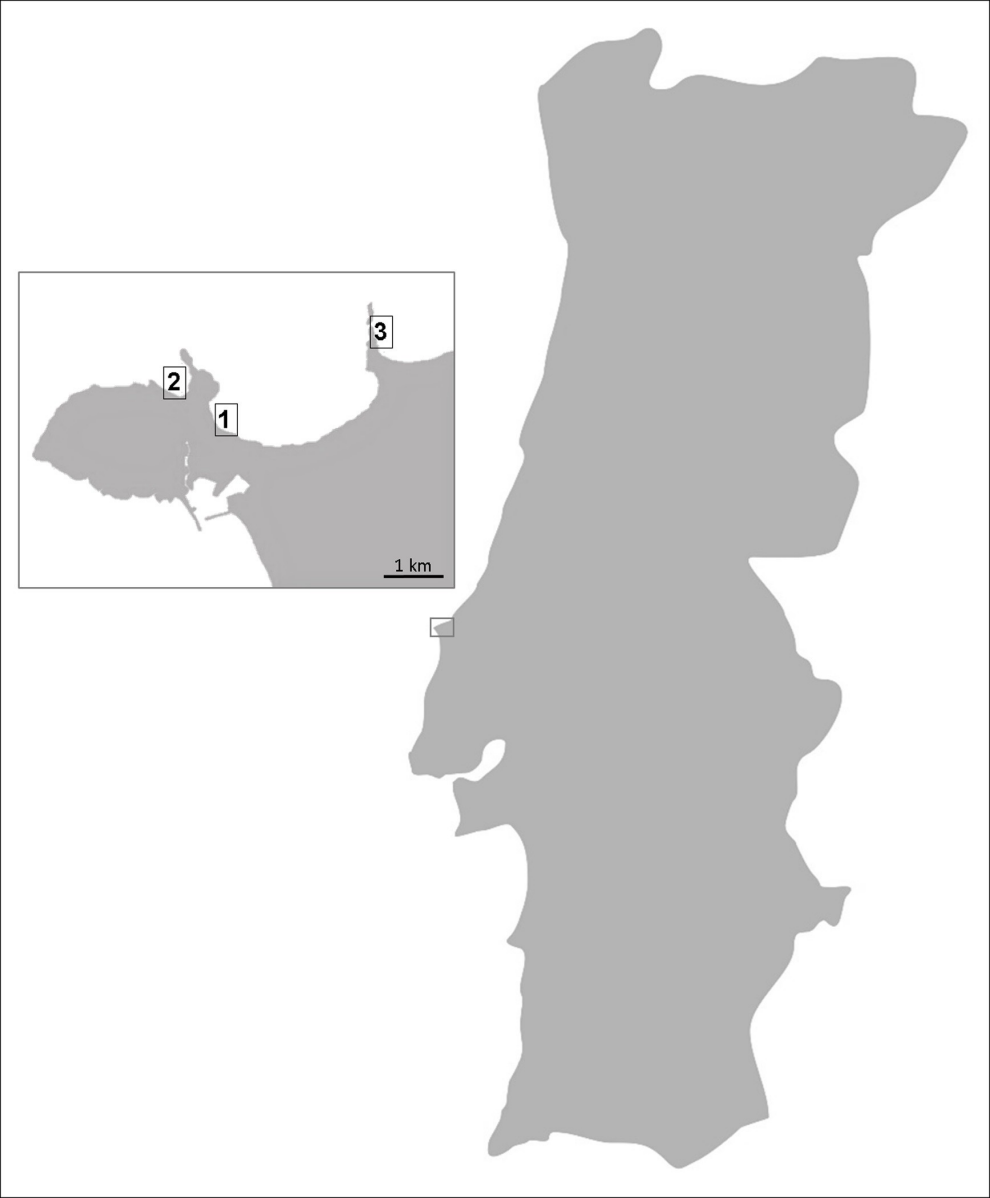
Collection areas: Gambôa (39.3651°N, 9.3728°W; 1); Portinho da Areia Norte (39.3691°N, 9.3779°W; 2); Baleal, praia dos Barcos (39.3766°N, 9.3402°W; 3).

Attached fresh thalli of the seaweed *H*. *scoparia* were randomly collected once in three different sandy beaches of the west coast of Portugal: Gambôa, Portinho da Areia Norte and Baleal (praia dos Barcos), in June 2018, August 2018 and December 2018, respectively (Fig 1). The collection of the seaweed samples did not require any specific permissions, considering that *H*. *scoparia* does not represent an endangered or vulnerable species and the sampling beaches are not protected areas. The samples were immediately transported and processed in the laboratory.

Specifically, seaweeds were roughly washed with seawater to remove adherent debris and sand, then surface sterilized by sequential immersion of the samples in sterile seawater, 70% ethyl alcohol (5 seconds) and twice in sterile seawater. The chosen sample was cut aseptically into small segments (< 1cm^3^) and direct-plated onto nutrient-rich-and -poor media, i.e. malt extract agar (MEA) and corn meal agar (CMA) respectively, supplemented with chloramphenicol. The use of two different media intended to maximize the number of surveyed fungal species, given the species-specific nutritional requirements. Following the recommendations of Zuccaro et al. [34] and Zhang et al. [31], some treated algal segments were pressed against the surface of the culture medium plates in order to create an imprint on the medium and test the efficacy of the seaweed surface sterilization process. Plates were incubated at 28°C, in the dark, until all colonising fungi emerged from the inner algal tissue. Each fungal strain was continuously transferred onto a new plate with potato dextrose agar medium (PDA) until a pure culture was obtained. Each fungal isolate was sub-cultured into three new PDA plates for further procedures.

### Molecular identification of isolated fungi

Each isolate was exclusively identified by molecular methods, which involved the extraction of genomic DNA by an optimized phenol-chloroform method adapted from Liu et al. [44] and amplification and sequencing of internal transcribed spacers 1 and 2 and intervening 5.8S of nuclear ribosomal DNA (ITS) using primers pair ITS5 and ITS4 [45]. Polymerase chain reaction (PCR) amplifications were performed in 50 μL reaction volumes, containing 0.4 mM of dNTP´s, 0.4 μM of each primer, 5 μL of 10xDreamTaq Green Buffer, 0.25 μL of DreamTaq DNA Polymerase (Thermo Fisher Scientific, Waltham, MA, USA) and 2.5 μL of genomic DNA. PCR was carried out on a thermocycler (Bio-Rad T100, Hercules, CA, USA) using the following parameters: initial denaturation for 3 min at 95°C, 34 cycles of 30 s at 95°C, 30 s at 55°C and 1 min at 72°C, then a final elongation step of 10 min at 72°C. Amplicons were purified through GeneJet PCR Purification Kit (Thermo Fisher Scientific, Waltham, MA, USA) and directly sent to STABVIDA (Lisbon, Portugal) for sequencing, using the same pair of PCR primers. Obtained ITS sequences were blasted against those available on the National Center for Biotechnology Information (NCBI) GenBank database and identified to the lowest taxonomic level possible, based on cut-off values of 97% of sequence similarity and 90% of sequence cover for species [46, 47]. Even though ITS region is recommended as the official and universal barcode for fungi [48], it is not variable enough for distinguishing species included in certain genera. For this reason, a secondary barcode for identifying doubtful sequences to species level was proposed by some authors and adopted in this study. Different primers were chosen to amplify each gene based on literature recommendations for each taxonomic group. Specifically: partial calmodulin (cal) protein-coding gene was amplified and sequenced using primers pairs CALDF1/ CALDR1, CMD5/ CMD6, or CALD-38F/ CALD-752R [49–51]; partial β-tubulin (tub) protein-coding gene was amplified and sequenced using primers pairs Bt2a/ Bt2b, or T1/ Bt2b [51–53]; partial translation elongation factor 1 -alpha (tef1-α) gene was amplified and sequenced using primers pairs EF1/ EF2, or EF1-728/ EF1-986 [54, 55]; and partial histone H3 (his3) gene was amplified and sequenced using primers pair CYLH3F/ H3-1bR [51]. The PCR conditions for amplification of all above mentioned genes sequences were similar to those applied for ITS amplification, except the annealing temperatures that were adjusted to optimise each primers pair efficiency. The obtained sequences were compared again with those available in the GenBank database and identified to the lowest taxonomic category possible. A multilocus phylogenetic analysis was additionally performed in MEGA-X software [56] for fungal isolates whose sequences demonstrated a high homology with those of different species within the same genus. Concretely, sequences of different genes/regions from isolates and closely related species were aligned and concatenated, and a phylogenetic tree based on a Maximum-likelihood (ML) analysis was constructed. The best-fit nucleotide substitution model was selected for analysis, based on the lowest Akaike information criterion value. A bootstrap analysis was performed with 1000 replicates to evaluate the support of each node.

### Small-scale fermentation and extraction of secondary metabolites

Small-scale fermentation assay followed the protocol suggested by Kjer et al. [57]. Concretely, 7–8 mycelial plugs were removed from the actively growing edge of each pure culture and inoculated in 100 mL of liquid Wickerham’s medium (0.3% yeast extract, 0.3% mal extract, 0.5% peptone, 1% glucose). These cultures were incubated at 28°C in static and dark conditions, for 21 to 45 days depending on the fungal growth. The metabolites produced by each fungus and released to the medium and/or retained on the mycelial biomass were extracted by ethyl acetate (EtOAc) [58]. The methodology included the following sequential procedures: interruption of fermentation process by adding EtOAc to the liquid culture (1:1); fragmentation of the mycelium and standing of the mixture for 24 h at room temperature; fungal cell lysis by mechanical homogenisation (Ystral X10/25, Ballrechten-Dottingen, Germany); vacuum filtration; separation of the organic fraction, and re-extraction from the aqueous fraction with 1/2 and 1/4 of the initial EtOAc volume; combination of the three EtOAc fractions and evaporation of the solvent under vacuum, at 40°C, in a rotary evaporator (Heidolph, Laborota 4000, Schwabach, Germany). The obtained dried crude extracts were kept at -20°C until further analyses.

### Evaluation of the biological activities of crude extracts

The fungal crude extracts were screened for their antioxidant, enzymatic inhibitory, inflammatory/anti-inflammatory, photoprotective and antimicrobial activities. The dried extracts were dissolved in DMSO to obtain a stock solution (20 mg/mL), which was used on most of the following assays. DMSO was used as vehicle control, in order to exclude the possible contribution of the DMSO to the obtained results.

#### Total phenolic content and antioxidant capacity

The antioxidant potential of crude extracts was inferred from the quantification of the total phenolic content (TPC), and evaluated through 2,2-diphenyl-1-picrylhydrazyl (DPPH) radical scavenging activity, ferric reducing antioxidant power (FRAP) and oxygen radical absorbance capacity (ORAC), following the methods described by Pinteus et al. [59] and Silva et al. [60].

TPC was determined by the Folin–Ciocalteu method, with slight modifications and adapted to a microplate scale. The reaction occurred during 1 h in the dark, and the absorbance was measured at 755 nm in a microplate reader (Synergy H1 Multi-Mode Microplate Reader, BioTek® Instruments, Winooski, VT, USA). Gallic acid was used as standard for the calibration curve (0.01–1 mg/mL), and TPC was expressed in milligrams of gallic acid equivalents per gram of dry extract (mg GAE/ g of extract).

The determination of DPPH radical scavenging activity of crude extracts involved the mixture of each extract with DPPH radical solution (0.1 mM), standing for 30 min in the dark and at room temperature, and measurement of absorbance at 517 nm in the microplate reader. The concentration of the extracts that caused a 50% decrease in the DPPH absorbance (EC_50_) was determined, and the results were presented as EC_50_ values plus the confidence intervals for 95%.

The FRAP assay involved the mixture of each extract with the FRAP reagent (0.3 M acetate buffer, pH 3.6; 10 mM of TPTZ in 40 mM HCl; 20 mM ferric solution, with FeCl_3_ at a ratio of 10:1:1), standing for 30 min in the dark and at 37°C, and measurement of absorbance at 593 nm in the microplate reader. FeSO_4_ was used as standard for the calibration curve, and the results were expressed as micromolar of FeSO_4_ equivalents per gram of dry extract (μM of FeSO_4_/ g of extract).

The ORAC method involved the mixture of each extract with fluorescein (70 nM), standing for 15 min at 37°C, addition of AAPH solution (12 mM), and measurement of the fluorescence (λ excitation: 458 nm; λ emission: 520 nm) immediately and every minute thereafter for 240 min in the microplate reader. Trolox was used as antioxidant standard for the calibration curve, and the results were expressed in micromol of Trolox equivalents per gram of dry extract (μmol TE/g of extract).

#### Enzymatic inhibitory capacity

The potential of the crude extracts to inhibit the activity of the enzymes responsible for the degradation of the components of extracellular matrix in dermis (hyaluronidase, collagenase and elastase) and for the production of melanin (tyrosinase) was evaluated through different methods.

The inhibition of hyaluronidase activity was determined according to the method described by Yahaya and Don [61], with slight modifications and adapted to microplate scale. Specifically, this method involved the following sequential procedures: mixture of each extract (200 μg/mL) with hyaluronidase (7 U/mL) and standing for 10 min at 37°C; addition of hyaluronic acid solution (0.03% in 300 mM sodium phosphate; pH 5.35 at 37°C) and standing for 45 min at 37°C; addition of acid albumin solution (0.1% bovine serum albumin in 24 mM sodium acetate and 79 mM acetic acid; pH 3.75 at 25°C) to precipitate the undigested hyaluronic acid, and standing for 10 min at room temperature. The absorbance of each mixture was then measured at 600 nm in the microplate reader. The hyaluronidase inhibitory activity of each crude extract was inferred from the hyaluronic acid that remained in the wells.

The inhibition of collagenase activity by crude extracts at 200 μg/mL was estimated using the EnzChek™ Gelatinase/Collagenase Assay Kit (# E12055, Invitrogen™, ThermoFisher Scientific) according to manufacturers’ instructions.

The inhibition of elastase activity by crude extracts at 200 μg/mL was estimated using the EnzChek™ Elastase Assay Kit (# E12056, Invitrogen™, ThermoFisher Scientific) according to manufacturers’ instructions. Epigallocatechin gallate (EGCG) was used as positive control to inhibit elastase, collagenase, and hyaluronidase activities, and the results were expressed as a percentage of control.

The interference of the extracts in the tyrosinase activity was assessed through the oxidation reaction of L-DOPA, based on the method described by Lee et al. [62] and Senol et al. [63], with slight modifications. This method involved the following sequential steps: mixture of each extract (200 μg/mL) with potassium phosphate buffer (0.5 mM, pH = 6.8) and L-DOPA (1mM) in 96-well microplates; addition of tyrosinase (100 U/mL) and standing for 5 min, in the dark, at 37°C; measurement of absorbance at 475 nm in the microplate reader, every minute for 15 min. Kojic acid was used as antioxidant standard, and the results were expressed as a percentage of control.

The extracts that considerably inhibited enzymes ´performances at 200 μg/mL were additionally tested at 100, 60, 30 and 10 μg/mL to determine the concentration that inhibited 50% of enzyme activity (IC_50_).

#### Anti-inflammatory and photoprotective capacities assessed on *in vitro* cell models

*Maintainance of cell cultures*. The anti-inflammatory and photoprotective activities of the crude extracts were evaluated *in vitro* on RAW 264.7 murine macrophages and 3T3 murine fibroblasts, respectively. The RAW 264.7cells were acquired from American Type Culture Collection (ATCC-TIB-71), and were cultured in Dulbecco´s Modified Eagle´s medium that contains Nutrient Mix F-12 supplemented with 10% fetal bovine serum, 1% antibiotic—antimycotic (amphotericin B, 0.25 mM; penicillin, 60 mM; streptomycin, 100 mM) and 1% sodium pyruvate. The 3T3 cells were acquired from German Collection of Microorganisms and Cell Cultures GmbH (DSMZ-ACC 173), and were cultured in the same medium, but without sodium pyruvate. Cells were kept in a 95% moisture and 5% CO_2_ at 37°C.

*Anti-inflammatory capacity*. The assessment of this biological activity involved a previous determination of the maximum concentrations of extracts that were innocuous to RAW 264.7 cells. This assay included the seeding of RAW 264.7 cells in 96-well microplates (5×10^4^ cells), incubation for 16 h and exposure of cells to various concentrations of different crude extracts for 24h. The cell viability was then assessed using the 3-[4, 5-dimethylthiazol-2-yl]-2, 5-diphenyltetrazolium bromide (MTT) colorimetric assay described by Mosmann [64], with slight modifications and adapted to microplate scale. Specifically, treated cells were incubated with MTT for 1 h, at 37°C, and the formazan crystals that resulted from the reduction of MTT by viable cells were solubilized in DMSO.

The inflammatory and anti-inflammatory activities of crude extracts were evaluated in RAW 264.7 cells through the levels of nitric oxide (NO) produced by these cells, following the methodology described by Yang et al. [65], with slight modifications. Briefly, the first screening involved the exposure of RAW 264.7 cells to the non-toxic concentrations of extracts for 24 h; and the second screening, the exposure of RAW 264.7 cells to the same non-toxic concentrations of the extracts for 1 h, and then to 1 μg/ mL of lipopolysaccharides (LPS) for 24 h. Cells only exposed to LPS (1 μg/mL) were used as a positive control. After the incubation, 100 μL of cell culture medium from each well were mixed with 100 μL of Griess reagent (1% (w/v) sulfanilamide, 0.1% (w/v) N-(1-naphthyl) ethylenediamine in 2.5% (v/v) phosphoric acid) and incubated for 15 min, in the dark, at room temperature. The quantity of nitrite that reflect the NO production was then measured at 546 nm in the microplate reader. The results were expressed as a percentage of control (untreated cells).

*Photoprotective capacity*. The assessment to this biological activitiy involved also a prior determination of the non-toxic concentrations of extracts to 3T3 cells. This assay included the following sequential steps: seeding and incubation of 3T3 cells in 96-well microplates until reached 90% confluence; exposure of cells to various concentrations of different crude extracts for 24h; and assessment of cell viability through the MTT colorimetric method [64].

The photoprotective activity of crude extracts was inferred from the capacity of the extracts to reduce the production of ROS by 3T3 cells, following the method described by Marto et al. [66], with slight modifications. Firstly, 3T3 cells were incubated with non-toxic concentrations of extracts for 1 h, in the dark, at 37°C. Secondly, treated cells were exposed to a UV radiation (12.5 mJ/cm^2^) for 1 h, in a UV curing chamber (UVA Cube 400, Hönle Technology, Gräfelfing, Germany). Finally, 100 μL of 2’,7’-dichlorodihydrofluorescein diacetate *(*H2-DCFDA, 20 mM) were added to cells, which were then incubated for 30 min, in the dark, at 37°C. The production of ROS was obtained from the fluorescence intensities (λ excitation: 495 nm; λ emission: 527 nm) measured immediately, and every minute thereafter for 10 min, in the microplate reader. The results were expressed as a percentage of control.

#### Antimicrobial capacity

The antimicrobial potential of the crude extracts was assessed against three different microorganims involved in skin disorders, i.e. two Gram-positive bacteria, *S*. *epidermidis* (DSM 1798) and *C*. *acnes* (DSM 1897), and one fungus, *Malassezia furfur* (DSM 6170), acquired from DSMZ biobank. The methodology followed the protocol described by Horta et al. [67], with minor adaptations. Firstly, the microorganisms were cultivated in the following conditions: *S*. *epidermidis* grown on Trypticase Soy Broth medium with 0.3% yeast extract, at 37°C; *C*. *acnes* grown on Tryptic Soy Broth medium with 5% sheep blood, in anaerobic conditions, at 37°C; and *Malassezia furfur* grown on Leeming-Notman Broth medium at 30°C. Microorganisms were then transferred to 96-well plates when an initial optical density (O.D) of 0.1 (5 μL) was achieved, and incubated with the fungal extracts at a concentration of 200 μg/mL (5 μL) in the same media previously indicated (193 μL). The inhibitory capacity of each extract was determined during the exponential growth of microorganisms, by measuring the O.D. at 600 nm, in the microplate reader. Oxytetracycline (VWR-BDH Chemicals-Prolabo, Leuven, Belgium) and amphotericin B (VWR-BDH Chemicals-Prolabo, Leuven, Belgium) were used as antibacterial and antifungal positive controls to inhibit the growth of *S*.*epidermis* and *C*. *acnes*, and *M*. *furfur*, respectively. The results were expressed as a percentage of control. The extracts that inhibited more than 50% of the microbial growth at 200 μg/mL, were additionally tested at 100, 60, 30 and 10 μg/mL to determine the IC_50_.

### Chemical characterization of fungal extracts

Crude extracts were screened regarding their absorption profile on the ultraviolet-visible (UV–Vis) region, while the chemical composition of the most bioactive extract was screened through high resolution tandem mass spectrometry (HRMS/MS).

#### UV–Vis absorption profiles

The crude extracts were dissolved in methanol (1 mg/mL), and their UV–Vis absorption spectra, in the wavelength range of 200–800 nm, were obtained on a UV-Vis spectrophotometer (Evolution 201,Thermo Scientific, Madison, WI, USA).

#### Tandem mass spectrometry (HRMS/MS) analysis

The tandem mass analysis was performed on a Bruker Impact II quadrupole time-of flight (QTOF) mass spectrometer equipped with an electrospray ionization (ESI) source (Bruker, Bremen, Germany), using the Data Analysis 4.4 software. A methanolic solution (500 μg/mL) of the most bioactive fungal extract was analysed by direct infusions. Spectra were obtained in the positive ESI (+) and negative ESI (-) modes in the *m/z* 50–1000 range. The capillary voltage was set to 4.5 kV and 3.5 kV for the negative and positive modes, respectively. The dry gas was kept at 4.0 L/ min, at 200°C. The quadrupole ion energy was set to 5.0 eV, while the collision cell energy was set to 10.0 eV. A flux of 200 μL/h was used. Before the sample being injected, the calibration was made using a standard solution which was prepared adding 250 mL water, 250 mL isopropanol, 750 μL acetic acid, 250 μL formic acid, and 500 μL NaOH solution (1 mol/L).The determination of the molecular formula of each metabolite and their corresponding ions fragments were based on the information provided by accurate mass and isotopic pattern of protonated molecules (precursors ions) and the peaks found in MS/MS spectra for each metabolite.

### Data and statistical analysis

The data obtained in this study were checked for normality and homoscedasticity and one-way analysis of variance (ANOVA) with Dunnett’s multiple comparison of group means to determine significant differences relatively to control treatment was performed. Kruskal–Wallis non-parametric tests were additionally carried out when assumptions of ANOVA were not met. At least three independent experiments were carried out in triplicate for most of the assays. The differences were considered significant at a level of 0.05 (*p* < 0.05) and the results are presented as mean ± standard error of the mean (SEM). The IC_50_ and EC_50_ were determined by the analysis of non-linear regression using GraphPad Prism software and the equation y = 100/ (1 + 10 ^(X − LogIC50/EC50)^). Calculations were performed using IBM SPSS Statistics 24 (IBM Corporation, Armonk, NY, USA) and GraphPad v5.1 (GraphPad Software, La Jolla, CA, USA) softwares.

## Results

### Endophytic fungal community associated with *Halopteris scoparia*

The molecular identification of the ten isolates recovered from *H*. *scoparia* revealed eight ascomycetous fungal taxa included in the classes *Eurotiomycetes*, *Sordariomycetes* and *Dothideomycetes* (Table 1).

**Table 1:** Endophytic fungi isolated from *Halopteris scoparia* and molecularly identified by comparison of ITS and cal, tef1-α, tub and/or his3 sequences with those available on GenBank database.

Collection area (date)	Isolate	Identified fungal taxa	NCBI accession n°	Discriminatory loci	Cover/ Identity (%)	BLAST best hits (NCBI accession n°)
Gb (Jun.18)	13	*Aspergillus chevalieri* ^a^	MW856073	cal	98/ 100	*Aspergillus chevalieri* (MK451332)
AN (Aug.18)	20	*Aspergillus chevalieri*	MW856074	cal	100/ 100	*Aspergillus chevalieri* (MK451332)
Bl (Dec.18)	93	*Penicillium sizovae* ^a^	MW856079	tub	99/ 100	*Penicillium sizovae* (LT898305)
Bl (Dec.18)	92	*Penicillium* sect. *Exilicaulis* ^a^	MW856078	tub	100/ 99	*Penicillium arabicum* (LT898226)
Gb (Jun.18)	1A	*Diaporthe* sp. ^b^	MW822676	ITS	100/100	*Diaporthe leucospermi* (JN712460)
			MW856070	tef1-α	99/ 98.5	*Diaporthe rossmaniae* (MK828064)
			MW856076	tub	97/ 99.4	*Diaporthe rossmaniae* (MK837915)
			MW856072	cal	76/ 99.8	*Diaporthe rossmaniae* (MK883823)
			MW856069	his3	100/ 99.8	*Diaporthe rossmaniae* (MK871433)
Gb (Jun.18)	2	*Nigrospora oryzae* ^b^	MW822677	ITS	100/ 99.6	*Nigrospora oryzae* (MH003399)
Bl (Dec.18)	89	*Nigrospora oryzae*	MW822678	ITS	100/ 100	*Nigrospora oryzae* (GU073124)
Bl (Dec.18)	90	*Fusarium incarnatum-equiseti* complex ^b^	MW856071	tef1-α	100/ 98.3	*Fusarium equiseti* (DQ854856)
Bl (Dec.18)	88	*Arthrinium arundinis* ^b^	MW856077	tub	99/100	*Arthrinium arundinis* (AB220321)
AN (Aug.18)	22	*Stemphylium gracilariae* ^c^	MW856075	cal	99/ 100	*Stemphylium gracilariae* (KU850839)

^a^ *Eurotiomycetes*,

^b^ *Sordariomycetes*,

^c^ *Dothideomycetes*.

* BLAST best hit for the ITS sequence of isolate 1A, considering that the results from BLAST varied slightly depending on the gene compared against the NCBI database. Collection areas are abbreviated as folllows: Gb, Gambôa; AN, Portinho da Areia Norte; Bl, Baleal.

The sequencing of second DNA barcodes from fungal isolates 13, 20, 93, 88 and 22 was fundamental for the identification to species level of *Aspergillus chevalieri*, *Penicillium sizovae*, *Arthrinium arundinis* and *Stemphyllium gracilariae*. On the other hand, and even though the high homology between ITS and second DNA barcode sequences of the isolate 90 and those available on GenBank, the high taxonomic complexity of genus *Fusarium* did not enable an accurate identification to species level. Similarly, the identification of isolate 90 was only possible to the section level given the high homology of ITS and β-tubulin sequences of different species included in section *Exilicaulis*. Also, and even though the process to identify the isolate 1A involved the construction of Maximum likelihood phylogenetic trees based on single and multi-locus alignments of the sequences of ITS region, and tef1-α, tub, cal and his3 genes of isolate 1A and closely related species as suggested by Santos et al. [68], the results were not discriminatory enough to provide an identification of the isolate to the species level (S1 and 2 Figs).

**Fig 2:**
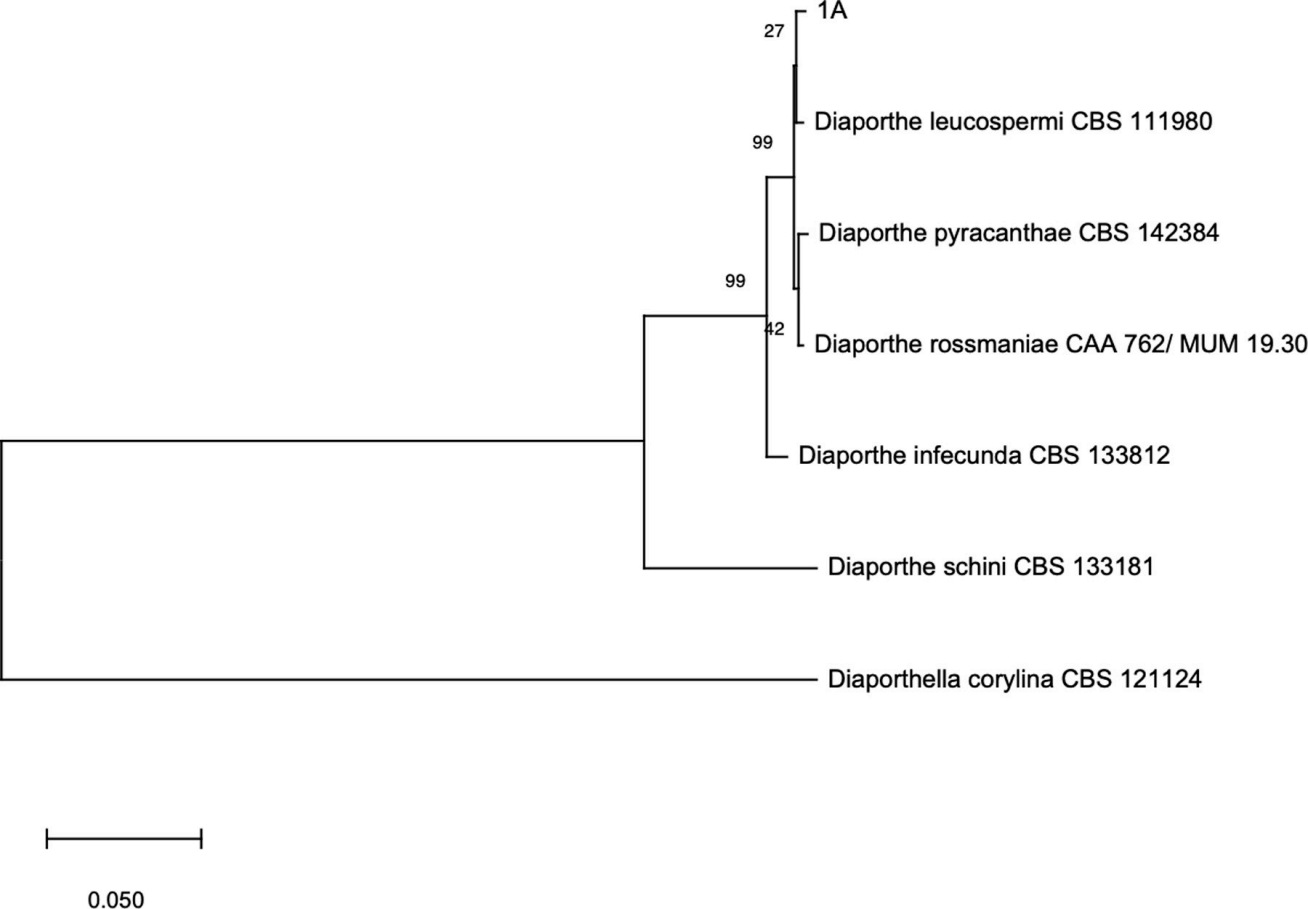
Maximum likelihood phylogenetic tree inferred from a concatenated dataset of ITS, tef1-α, β-tubulin, cal and his3 sequences of isolate 1A and BLAST best hits. *Diaporthella corylina* was used as outgroup. ML analysis was based on the Tamura-Nei model [69] and the tree was obtained from an initial bio-neighbour-joining tree automatically generated by the software, followed by a heuristic search using the nearest-neighbour interchange algorithm. A discrete gamma distribution was used to model evolutionary rate differences among sites. The percentage of trees in which the associated taxa clustered together is shown next to the branches.

These results indicate that the identification of *Penicillium* sect. *Exilicaulis*, *Fusarium incarnatum-equiseti* complex and *Diaporthe* sp. will require a further observation of the morphology of reproductive structures differentiated by each fungus in culture, as well as morphological features of the culture themselves in particular media.

The results also revealed that most of the fungi were isolated only once, while *A*. *chevalieri* and *Nigrospora oryzae* were collected in two different areas and sampling periods (Table 1).

### Biological activities of fungal crude extracts

The screening of secondary metabolites produced by different marine endophytic fungi under the same fermentation conditions for antioxidant, enzymatic inhibitory, anti-inflammatory and photoprotective effects showed that each fungal crude extract exhibited one or more relevant biological activities.

#### Antioxidant capacity

The combination of the results obtained by DPPH, FRAP and ORAC methods revealed that all the fungal extracts displayed antioxidant capacities; for some of the extracts, this biological activity is correlated with the total phenolic content (Table 2).

**Table 2:** Total phenolic content and antioxidant capacity of the crude extracts obtained from marine endophytic fungi associated with *Halopteris scoparia*.

Fungal producers	TPC^a^	DPPH^b^	FRAP^c^	ORAC^d^
*Aspergillus chevalieri* (isolate 13)	402.9 ± 42.0	35.5 (32.5–38.8)	3256.1 ± 174.3	19870.6 ± 1275.8
*Aspergillus chevalieri* (isolate 20)	191.2 ± 9.4	39.7 (34.3–46.0)	509.5 ± 36.5	1319.6 ± 198.5
*Penicillium sizovae*	33.0 ± 1.3	>200	498.6 ± 22.2	2058.9 ± 284.5
*Penicillium* sect. *Exilicaulis*	28.8 ± 2.4	>200	452.8 ± 68.3	1070.9 ± 81.0
*Diaporthe* sp.	20.2 ± 1.7	>200	215.2 ± 10.1	2823.0 ± 118.6
*Nigrospora oryzae* (taxon 2)	18.0 ± 1.4	>200	263.6 ± 9.5	2766.5 ± 313.2
*Nigrospora oryzae* (taxon 89)	31.0 ± 0.9	>200	494.8 ± 27.0	2814.1 ± 226.7
*Fusarium incarnatum-equiseti* complex	49.5 ± 2.6	>200	280.8 ± 43.1	2579.9 ± 151.6
*Arthrinium arundinis*	8.1 ± 0.6	>200	33.2 ± 3.7	441.4 ± 42.5
*Stemphylium gracilariae*	22.7 ± 3.6	>200	70.7 ± 7.2	870.4 ± 107.2
BHT	-	164.5 (142.7–189.7)	2821.5 ± 51.5	142.9 ± 8.7

^a^ Gallic acid equivalents per g extract (mg GA/g);

^b^ Radical scavenging activity (EC_50_ μg/mL);

^c^ FeSO_4_ equivalents per g extract (μM FeSO_4_/g);

^d^ Trolox equivalents per g extract (μmol TE/g). EC_50_ values were determined for a 95% confidence interval. The synthetic antioxidant butylated hydroxytoluene (BHT) was used as a reference compound.

Moreover, the results were concordant in identifying the fungal extracts with higher and lower antioxidant activities. Specifically, the extracts obtained from *A*. *chevalieri* and, particularly, the one from isolate 13 exhibited the highest antioxidant abilities among all the tested extracts and reference compound butylated hydroxytoluene (BHT), by showing lower half-maximal effective concentrations (EC_50_) for scavenging DPPH free radicals (35.5 μg/ mL) and higher values of TPC (402.9 mg GA/g), FRAP (3256.1 μM FeSO_4_/g) and ORAC (19870.6 μmol TE/g). On the other hand, the extract obtained from *A*. *arundinis* revealed the lowest capacity for scavenging DPPH free radicals at 200 μg/mL and lowest values of TPC (8.1 mg GA/g), FRAP (33.2 FeSO_4_/g) and ORAC (441.4 μmol TE/g).

#### Enzymatic inhibitory capacity

**Fig 3:**
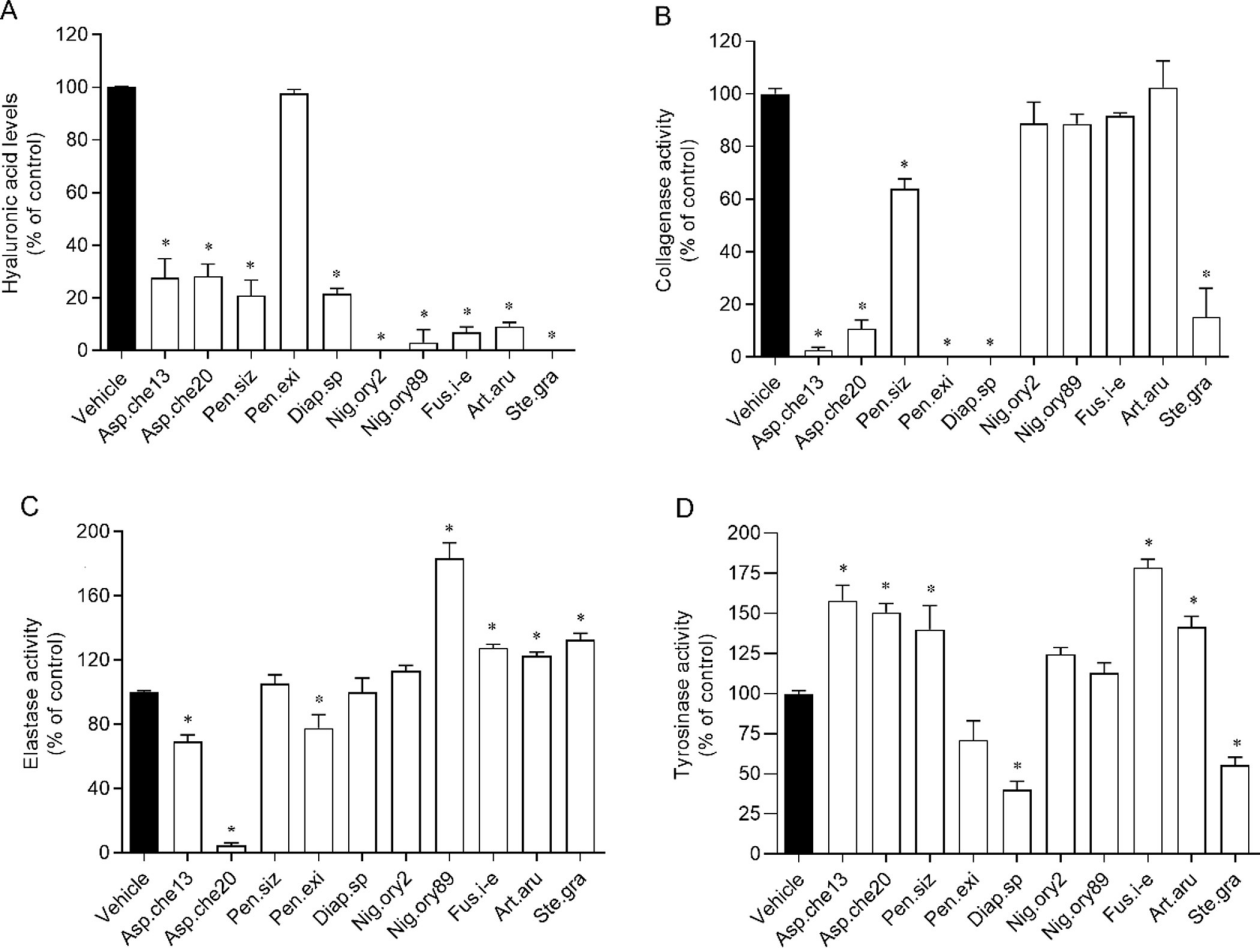
Inhibitory activity of the crude extracts obtained from endophytic fungi associated with *Halopteris scoparia* against hyaluronidase (A), collagenase (B), elastase (C) and tyrosinase (D) activities: inhibition of hyaluronidase is represented by the percentage of hyaluronic acid that was not degraded in the presence of 200 μg/mL of each extract; and inhibition of collagenase, elastase, and tyrosinase are represented by the interference of 200 μg/mL of each extract on their activity. The fungi from which the extracts were obtained are abbreviated as follows: Asp.che13 or 20, isolates 13 or 20 of *Aspergillus chevalieri*; Pen.siz, *Penicillium sizovae*; Pen.exi, *Penicillium* sect. *Exilicaulis*; Diap.sp, *Diaporthe* sp.; Nig.ory2 or 89, isolates 2 or 89 of *Nigrospora oryzae*; Fus.i-e, *Fusarium incarnatum-equiseti* complex; Art.aru, *Arthrinium arundinis*; Ste.gra, *Stemphylium gracilariae*. The values correspond to mean ± SEM of 3 independent experiences, in duplicate. * marks significant differences (One-way ANOVA, Dunnett’s test; *p*< 0.05) when compared with the vehicle.

The results showed that most of the tested fungal extracts exerted inhibitory effects on hyaluronidase, collagenase and/or elastase activities (Fig 3 and Table 3).

**Table 3:** Concentration of the fungal crude extracts that causes 50% inhibition of an enzymatic reaction (IC_50_).

Fungal producers	Hyaluronidase (μg/mL)	Collagenase (μg/mL)	Elastase (μg/mL)	Tyrosinase (μg/mL)
*Aspergillus chevalieri* (isolate 13)	>200	65.6 (79.7–115.5)	>200	>200
*Aspergillus chevalieri* (isolate 20)	>200	51.7 (37.8–69.7)	56.1 (48.5–64.5)	>200
*Penicillium sizovae*	>200	>200	>200	>200
*Penicillium* sect. *Exilicaulis*	99.1 (87.3–112.6)	107.0 (74.0–116.9)	>200	>200
*Diaporthe* sp.	>200	98.6 (86.7–120.2)	>200	39.8 (32.2–49.1)
*Nigrospora oryzae* (taxon 2)	>200	>200	>200	>200
*Nigrospora oryzae* (taxon 89)	>200	>200	>200	>200
*Fusarium incarnatum-equiseti* complex	>200	>200	>200	>200
*Arthrinium arundinis*	>200	>200	>200	>200
*Stemphylium gracilariae*	>200	112.3 (90.6–142.0)	>200	>200
EGCG	119.1 (126.1–320.4)	4.8 (4.1–5.5)	113.9 (80.7–160.0)	-
Kojic acid	-	-	-	18.3 (14.0–23.9)

The majority of fungal extracts interfered on hyaluronidase activity, by reducing slightly its activity (isolate 89 of *N*. *oryzae*< *Fusarium incarnatum*-*equiseti* complex< *A*. *arundinis*< *P*. *sizovae*< *Diaporthe* sp.< *A*. *chevalieri*) to almost completely (*Penicillium* sect. *Exilicaulis*) at 200 μg/mL (Fig 3A). The crude extract biosynthesised by *Penicillium* sect. *Exilicaulis* presented an IC_50_ of 99.1 μg/mL, which was slightly inferior to the IC_50_ of positive control (Table 3). On the other hand, the extracts from *S*. *gracilariae* and isolate 2 of *N*. *oryzae* did not show any inhibitory effect on hyaluronidase activity (Fig 3A).

Concerning collagenase activity, most of the extracts demonstrated an ability to inhibit partially or completely its activity at 200 μg/mL (Fig 3B). The extracts from *S*. *gracilariae*, *Penicillium* sect. *Exilicaulis*, *Diaporthe* sp. and isolates 13 and 20 of *A*. *chevalieri* that revealed higher inhibitory potencies were additionally tested at lower concentrations, revealing IC_50_ values of 112.3 μg/mL, 107.0 μg/mL, 98.6 μg/mL, 65.6 μg/mL and 54.3 μg/mL respectively (Table 3). The extracts from *N*. *oryzae*, *Fusarium incarnatum*-*equiseti* complex and *A*. *arundinis* did not exert a significant effect on collagenase activity (Fig 3B).

Regarding elastase activity, the results revealed that only the extracts from *A*. *chevalieri* and *Penicillium* sect. *Exilicaulis* interfered on its performance (Fig 3C). The extract of isolate 20 inhibited almost completely the elastase activity, presenting an IC_50_ of 56.1 μg/mL; this value was considerably lower than the IC_50_ of positive control (Table 3). The extracts from isolate 13 and *Penicillium* sect. *Exilicaulis* induced a decrease of c.a. 30%, at 200 μg/mL (Fig 3C). In contrast, and unexpectedly, the extracts from isolate 89 of *N*.*oryzae*, *Fusarium incarnatum*-*equiseti* complex, *A*. *arundinis*, and *S*. *gracilariae* promoted a significant increase of elastase activity.

The tyrosinase activity was strongly inhibited by the extract of *Diaporthe* sp., which showed an IC_50_ of 39.8 μg/mL (Table 3), and moderate-reduced by the extract of *S*. *gracilariae* (Fig 3D). The extracts from both isolates of *N*. *oryzae* and *Penicillium* sect. *Exilicaulis* did not display any effect, while the remaining extracts induced an increase of the hydrolysis carried out by tyrosinase.

Considering all the results together, the extracts provided by *A*. *chevalieri and Penicillium* sect. *Exilicaulis* presented the highest inhibitory potential against the majority of tested enzymes, whereas the extract of isolate 2 of *N*. *oryzae* was the only one without any inhibitory effect on the activity of hyaluronidase, collagenase, elastase and tyrosinase.

#### Inflammatory and anti-inflammatory capacities

**Fig 4:**
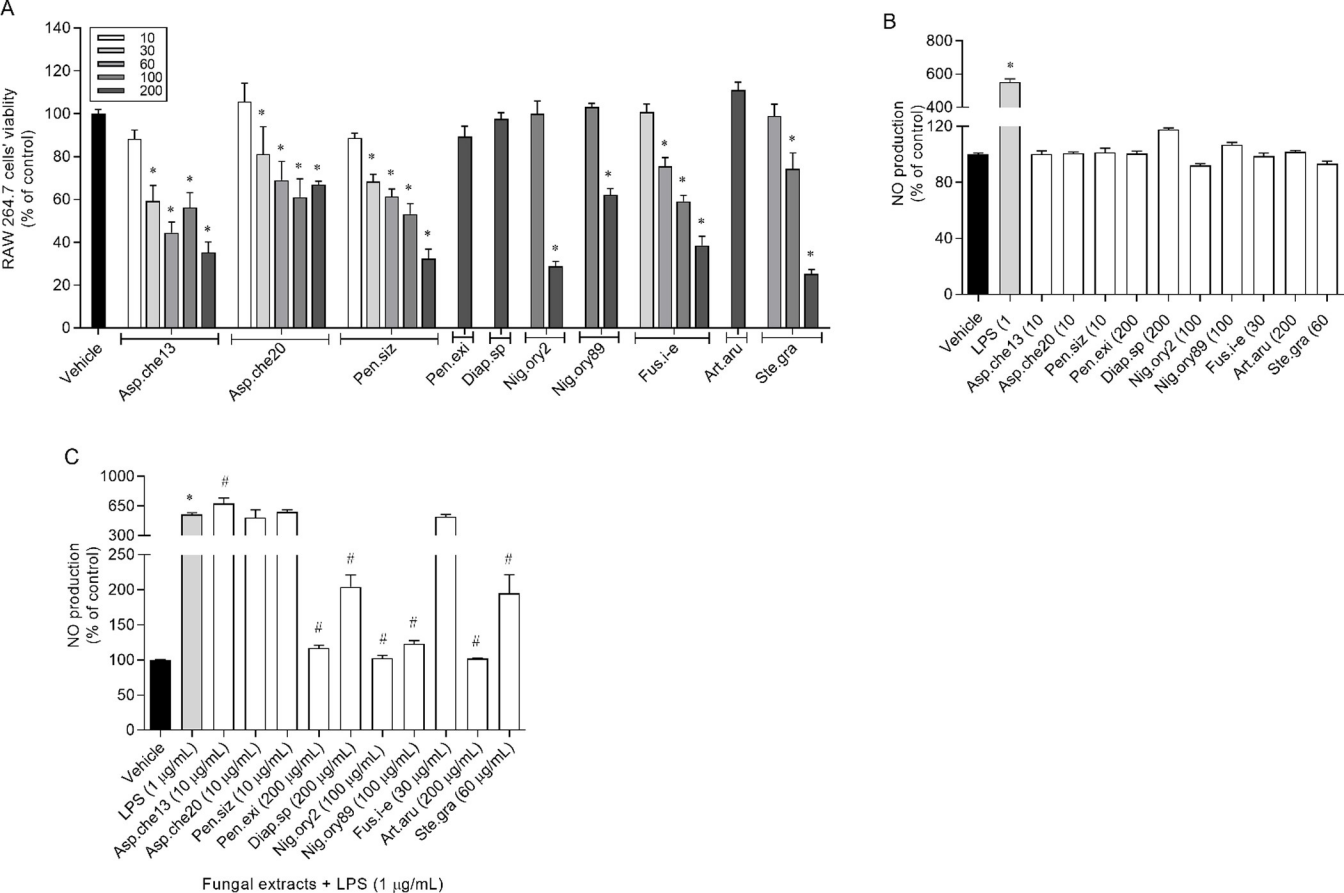
RAW 264.7 cells’ viability following 24 h of exposure to the crude extracts obtained from endophytic fungi associated with *Halopteris scoparia* (10–200 μg/ mL) and expressed as % of the control (A). Nitric oxide (NO) production induced on RAW 264.7 cells by fungal extracts at non-toxic concentrations after 24 h of treatment (B). Nitric oxide (NO) production induced on RAW 264.7 cells by fungal extracts at non-toxic concentrations and 1 μg/mL of LPS (C). The fungi from which the extracts were obtained are abbreviated as follows: Asp.che13 or 20, isolates 13 or 20 of *Aspergillus chevalieri*; Pen.siz, *Penicillium sizovae*; Pen.exi, *Penicillium* sect. *Exilicaulis*; Diap.sp, *Diaporthe* sp.; Nig.ory2 or 89, isolates 2 or 89 of *Nigrospora oryzae*; Fus.i-e, *Fusarium incarnatum-equiseti* complex; Art.aru, *Arthrinium arundinis*; Ste.gra, *Stemphylium gracilariae*. The values correspond to mean ± SEM. * and ^#^ represent significant differences (One-way ANOVA, Dunnett’s test; *p*< 0.05) when compared with the vehicle and LPS, respectively.

The screening of anti-inflammatory activities of fungal extracts required a previous determination of the concentrations at which the crude extracts were non-toxic to the RAW 264.7 cells. Those concentrations varied considerably among extracts (Fig 4A).

Specifically, the extracts from *Penicillium* sect. *Exilicaulis*, *Diaporthe* sp. and *A*. *arundinis* were non-toxic to RAW 264.7 cells at the highest tested concentration (200 μg/mL), and those of isolates 13 and 20 of *A*. *chevalieri* and *P*. *sizovae*, at the lowest concentration (10 μg/mL). The extracts from both isolates of *N*. *oryzae*, *S*. *gracilariae* and *Fusarium incarnatum-equiseti* complex were non-toxic to cells at concentrations of 100, 60 and 30 μg/mL, respectively.

The evaluation of the inflammatory effect of extracts at non-toxic concentrations on RAW 264.7 cells showed that none of the extracts stimulated significantly the production of NO (Fig 4B).

Moreover, the extracts from *Penicillium* sect. *Exilicaulis*, *Diaporthe* sp., both isolates of *N*. *oryzae*, *A*. *arundinis* and *S*. *gracilariae* demonstrated an ability to suppress significantly the NO production by RAW 264.7 cells when exposed to LPS (Fig 4C). The highest anti-inflammatory activity was mediated by the extracts from isolate 13 of *N*. *oryzae* and *A*. *arundinis*, considering that NO levels were reduced to basal values.

#### Photoprotective capacity

**Fig 5:**
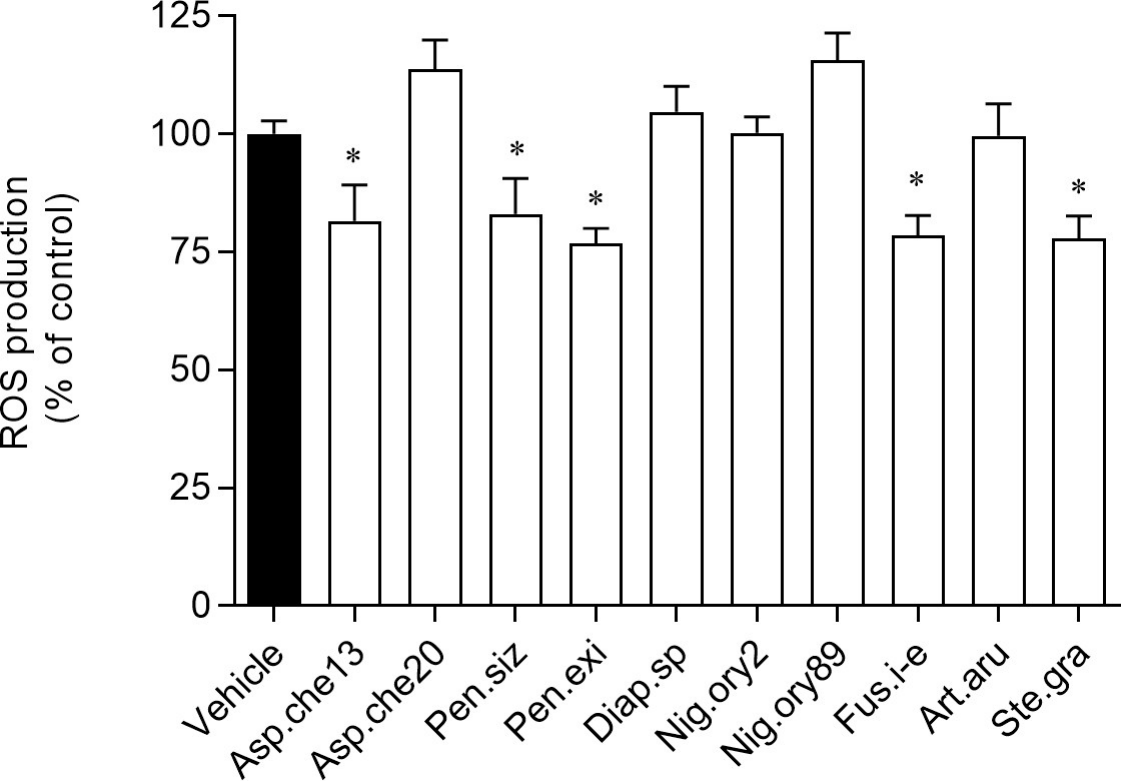
Reactive oxygen species (ROS) production induced on 3T3 cells after being treated with fungal extracts at 1 μg/mL and exposed to UV radiation (12.5 mJ/cm2). The fungi from which the extracts were obtained are abbreviated as follows: Asp.che13 or 20, isolates 13 or 20 of *Aspergillus chevalieri*; Pen.siz, *Penicillium sizovae*; Pen.exi, *Penicillium* sect. *Exilicaulis*; Diap.sp, *Diaporthe* sp.; Nig.ory2 or 89, isolates 2 or 89 of *Nigrospora oryzae*; Fus.i-e, *Fusarium incarnatum-equiseti* complex; Art.aru, *Arthrinium arundinis*; Ste.gra, *Stemphylium gracilariae*. The values correspond to mean ± SEM. * represents significant differences (One-way ANOVA, Dunnett’s test; *p*< 0.05) when compared with the vehicle.

The screening of photoprotective properties of the fungal extracts implied a previous determination of the maximum concentrations at which the extracts were non-toxic to fibroblasts (3T3). The results revealed that all the extracts were non-toxic at 1 μg/mL (S2 Fig), and thus, that concentration was used to test the capacity of the extracts to reduce the ROS production when cells were exposed to UV radiation. Extracts from isolate 13 of *A*. *chevalieri*, *P*. *sizovae*, *Penicillium* sect. *Exilicaulis*, *Fusarium incarnatum-equiseti* complex and *S*. *gracilariae* exhibited mild photoprotective abilities (Fig 5).

#### Antimicrobial capacity

**Fig 6:**
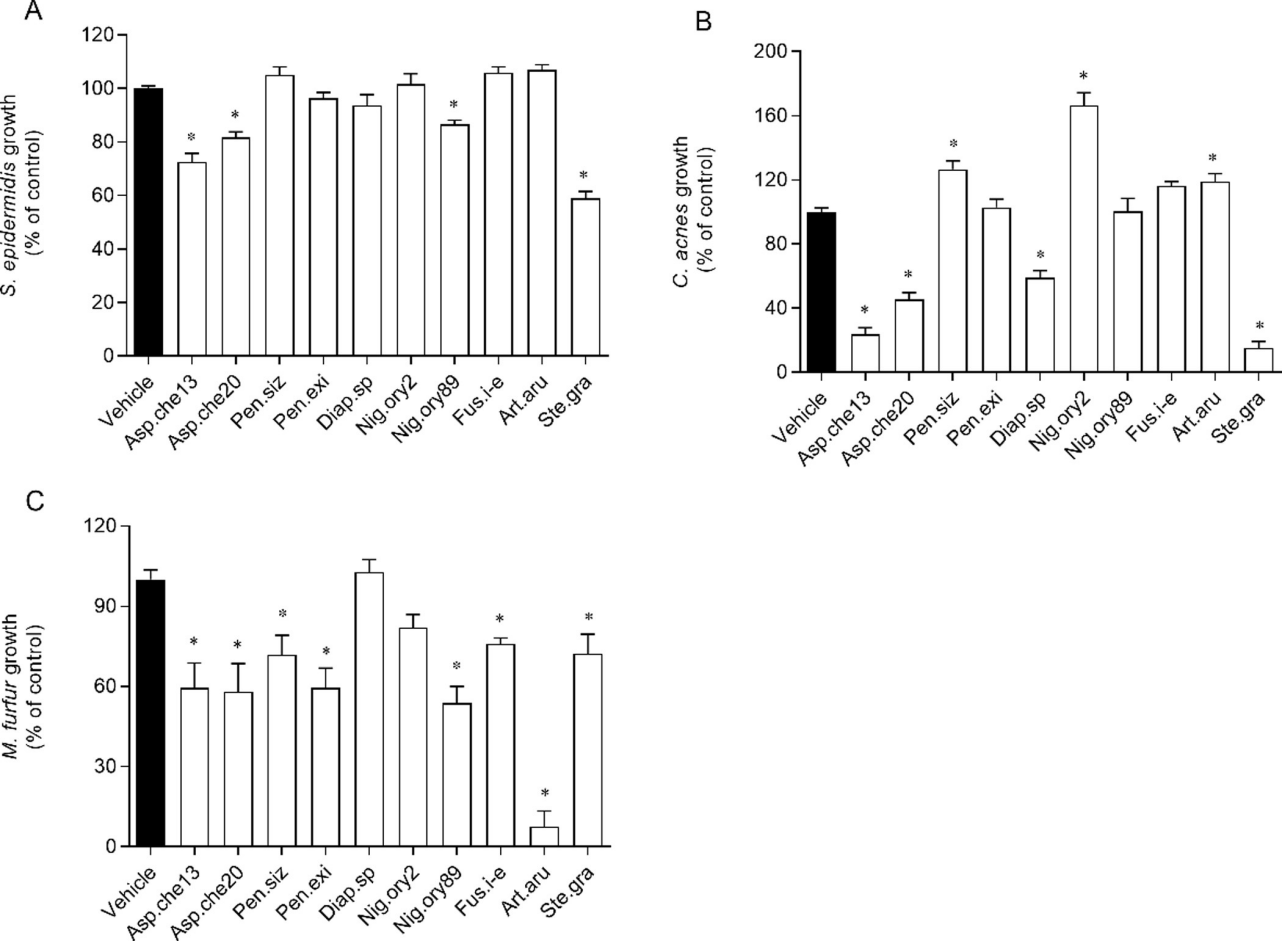
Inhibitory activity of the crude extracts obtained from endophytic fungi associated with *Halopteris scoparia* against *Staphylococcus epidermidis* (A), *Cutibacterium acnes* (B) and *Malassezia furfur* (C) growth, at 200 μg/mL. The fungi from which the extracts were obtained are abbreviated as follows: Asp.che13 or 20, isolates 13 or 20 of *Aspergillus chevalieri*; Pen.siz, *Penicillium sizovae*; Pen.exi, *Penicillium* sect. *Exilicaulis*; Diap.sp, *Diaporthe* sp.; Nig.ory2 or 89, isolates 2 or 89 of *Nigrospora oryzae*; Fus.i-e, *Fusarium incarnatum-equiseti* complex; Art.aru, *Arthrinium arundinis*; Ste.gra, *Stemphylium gracilariae*. The values correspond to mean ± SEM. * marks significant differences (One-way ANOVA, Dunnett’s test; *p*< 0.05) when compared with the vehicle.

The evaluation of antimicrobial potential of the crude extracts demonstrated that most of them inhibited the growth of one or more microorganisms (Fig 6 and Table 4).

**Table 4:** Concentration of the fungal crude extracts that causes 50% inhibition of microbial growth (IC_50_).

Fungal producers	*S*. *epidermidis* (μg/mL)	*C*. *acnes* (μg/mL)	*M*. *furfur* (μg/mL)
*Aspergillus chevalieri* (isolate 13)	>200	100.6 (89.9–125.1)	>200
*Aspergillus chevalieri* (isolate 20)	>200	152.3 (124.3–190.4)	>200
*Penicillium sizovae*	>200	>200	>200
*Penicillium* sect. *Exilicaulis*	>200	>200	>200
*Diaporthe* sp.	>200	>200	>200
*Nigrospora oryzae* (taxon 2)	>200	>200	>200
*Nigrospora oryzae* (taxon 89)	>200	>200	>200
*Fusarium incarnatum-equiseti* complex	>200	>200	>200
*Arthrinium arundinis*	>200	>200	25.6 (15.9–38.1)
*Stemphylium gracilariae*	>200	126.1 (110.2–142.3)	>200
Oxytetracycline	12.4 (11.2–16.1)	0.07 (0.05–0.09)	_
Amphotericin B	_	_	11.4 (8.6–15.0)

In a general perspective, the extracts from isolates 13 and 20 of *A*. *chevalieri* and *S*. *gracilariae* were the only extracts that exhibited antibacterial and antifungal properties; however, these extracts were more potent against *C*. *acnes* than *S*. *epidermidis* and *M*. *furfur*.

Additionally, the growths of *S*. *epidermidis* and *C*. *acnes* were slightly inhibited by the extracts from isolate 89 of *N*. *oryzae* and *Diaporthe* sp., respectively; and the growth of *M*. *furfur* was weakly inhibited by the extracts from *P*. *sizovae*, *Penicillium* sect. *Exilicaulis*, isolate 89 of *N*. *oryzae* and *Fusarium incarnatum-equiseti* complex, and strongly, by extracts from *A*. *arundinis* (IC_50_ of 25.6 μg/mL).

Even though the IC_50_ values of the extracts with more relevant antimicrobial effects may seem high when directly compared with IC_50_ of antibiotics, it is very likely that these values will be lower if the compounds responsible for antimicrobial activity are isolated and tested thereafter.

### Chemical characterization of fungal extracts

#### UV–visible absorption profiles

**Fig 7:**
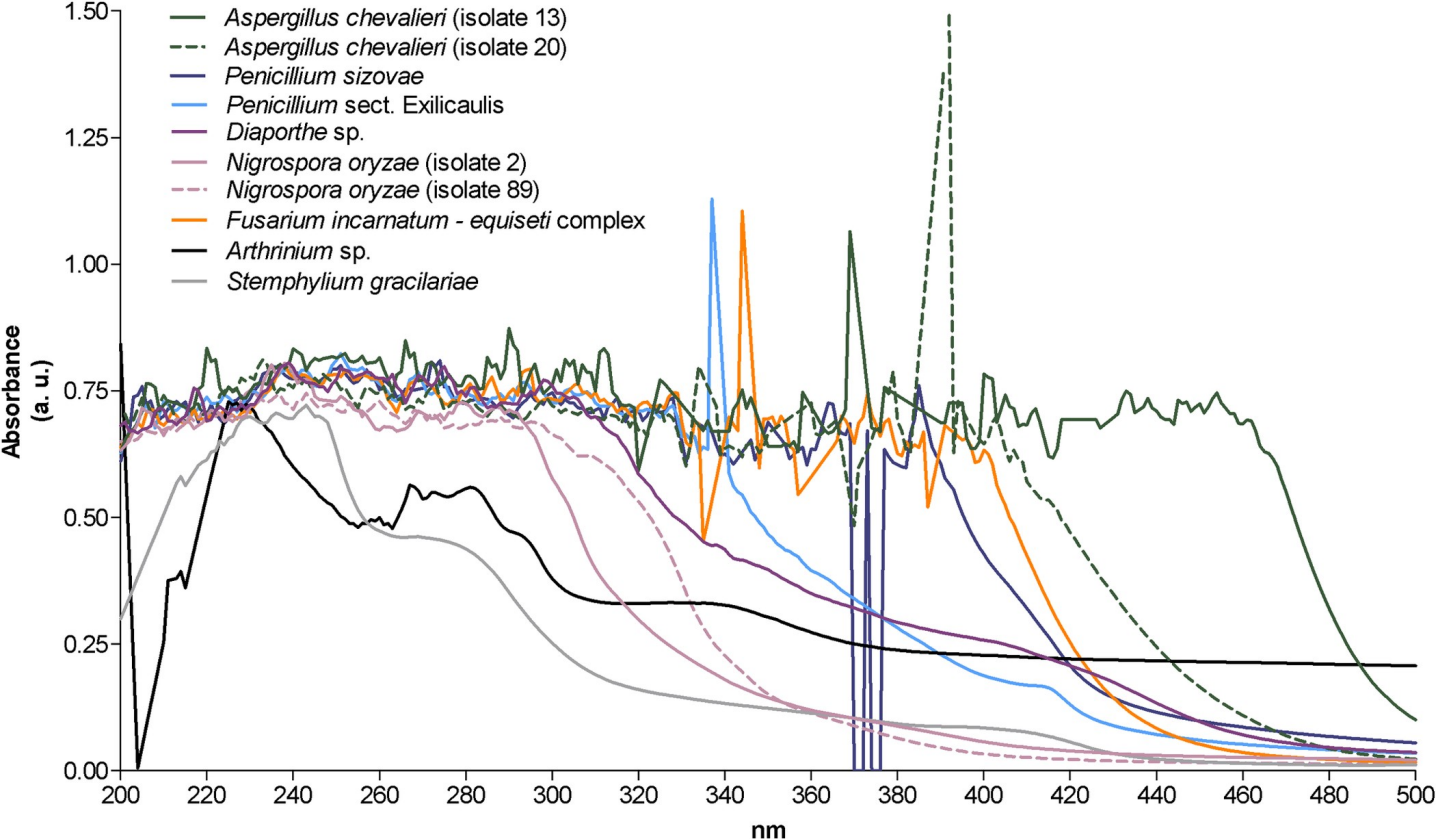
UV–Vis absorption spectra (200–500 nm) of the crude extracts obtained from marine endophytic fungi associated with *Halopteris scoparia*.

The UV-visible absorption spectra obtained for the fungal crude extracts showed significant absorbances in the UV-C (200–280 nm) and/or UV-B (280–320 nm) regions (Fig 7).

Besides noticeable absorbances in the UV-C and UV-B regions, the extracts from *Penicillium* sect. *Exilicaulis*, *Fusarium incarnatum-equiseti* complex, *P*. *sizovae*, and isolates 13 and 20 of *A*. *chevalieri* also exhibited strong absorbance in the UV-A region (320–400 nm), with absorption maxima at 337 nm, 344 nm, 385 nm, and 369 nm and 392 nm, respectively (Fig 7). The extract of isolate 13 also revealed additional absorbance peaks in the visible region (400–460 nm) of the spectrum (Fig 7).

#### Tandem mass spectrometry analysis of the most bioactive extract

**Fig 8:**
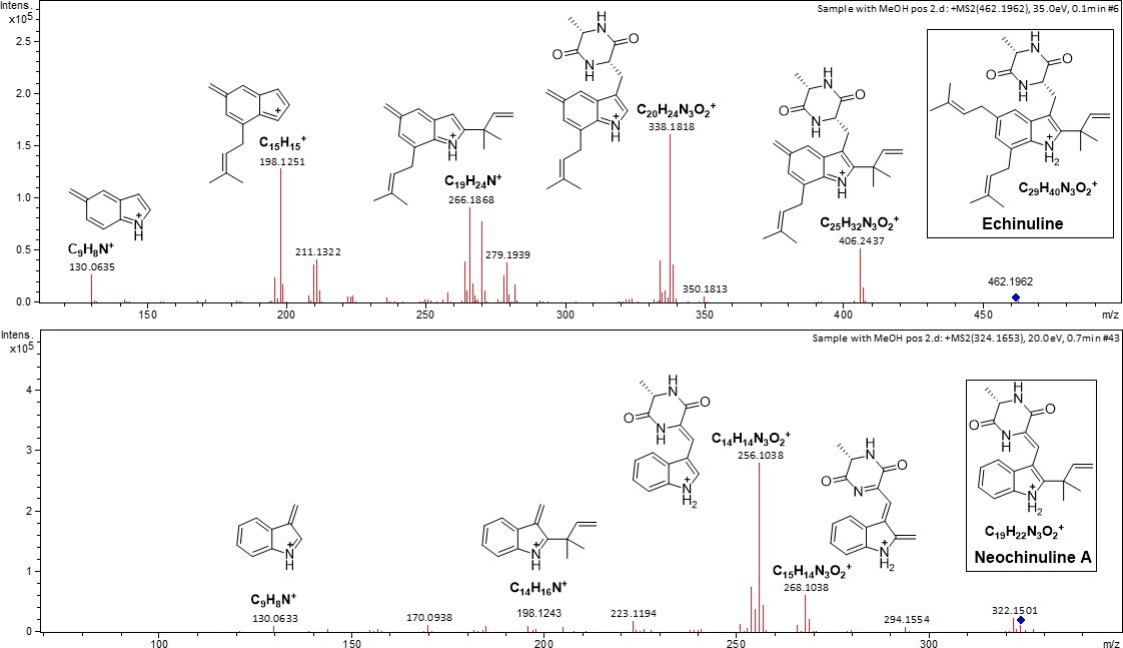
MS^2^ spectra of metabolites echinulin and neoechinulin A found in the crude extract from *Aspergillus chevalieri* (isolate13) by tandem mass spectrometry (HRMS/MS) analysis in ESI positive mode.

Based on all the results presented above and, specifically, the high number and potency of biological activities displayed by the crude extract from isolate 13 of *A*. *chevalieri*, this extract was selected for a HRMS/MS analysis. The data obtained in the ESI(+) mode allowed the identification of the natural products echinulin and neoechinulin A, by the presence in the MS spectra of the protonated molecules at *m*/*z* 462.1962 (C_29_H_40_N_3_O_2_^+^) and *m*/*z* 322.1501(C_19_H_22_N_3_O_2_^+^), and the corresponding fragmentation patterns found in MS^2^ spectra (Fig 8).

## Discussion

### Endophytic fungal community associated with *Halopteris scoparia*

The isolation and molecular-based identification methods used in this study revealed eight endophytic fungal taxa associated with *H*. *scoparia*. Nevertheless the fact that these fungi may not represent the most frequent and/or dominant species or the entire endophytic fungal community associated with this seaweed but probably are the most easily cultivable strains under laboratory conditions, this information contributes to a better understanding of the ecology of each colonising fungus. In reality, and though all the identified fungal species are known as generalist halo/osmotolerant fungi that occurred on both terrestrial and marine environments, this seems to be the first record of these species as endophytes of seaweeds and/or of *H*. *scoparia*. Concretely: *A*. *arundinis* was also isolated as endosymbionts of seaweeds *Sargassum fulvellum*, *Flabellia petiolata*, *Padina pavonica* and an unidentified seaweed, and from the halophyte *Salicornia europaea*, seagrass *Posidonia oceanica* and marine sponge *Phakellia fusca* [16, 36, 39, 70–73]; *A*. *chevalieri*, from marine sponges *Tethya aurantium* and *Grantia compressa*, and a coral *Anthozoa* sp.[74–76]; *N*. *oryzae*, from marine gorgonian *Verrucella umbraculum* and marine sponge *Phakellia fusca* [77, 78]; and *S*. *gracilariae*, from the seaweed *Gracilaria* sp. [79, 80]. *Arthrinium arundinis* was additionally identified on seawater and beach sand [16, 81], and *P*. *sizovae*, on hypersaline waters of salterns [82]. Moreover, the genera *Aspergillus*, *Fusarium*, *Penicillium* and *Stemphillium* identified in this study have also been frequently reported in fungal communities associated with seaweeds, particularly the genus *Aspergillus* [31, 72, 83–87].

The presence and dominance of fungal species in endophytic communities that are able to colonise marine and terrestrial environments and/or are associated with taxonomic distant hosts have been raising doubts among the scientific community concerning the ecological relationship of these fungi with the marine environments and their hosts. However, and taking into account that all the collected seaweeds in this study did not demonstrate any evidence of disease, it is very likely that these endophytic fungi are physiologically adapted and metabolically active in marine environments, and have established an endosymbiosis with their hosts that seems beneficial for both partners. Moreover, and following the hypothesis raised by Suryanarayanan et al. [35], the apparent loose host affiliation might be explained as an adaptation strategy to thrive under more stressful environmental conditions. Nevertheless this apparent non-host specificity, the isolation of *A*. *chevalieri* and *N*. *oryzae* from *H*. *scoparia* in two different areas and sampling periods in the present study also suggested that at least one of the partners might have some degree of preference or specificity for that ecological relationship.

### Biological activities of fungal extracts

**Fig 9:**
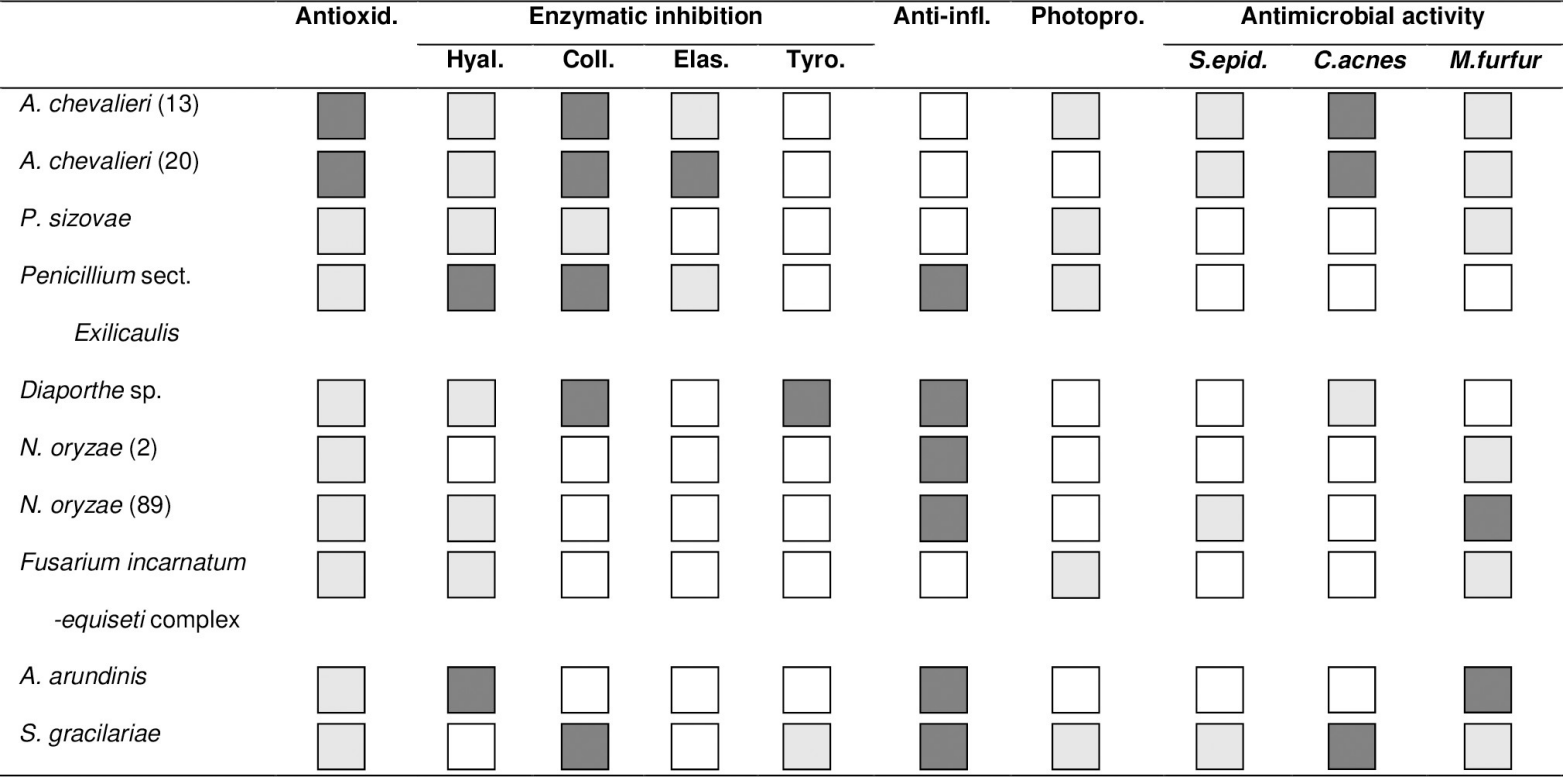
Overall overview of antioxidant, enzymatic inhibitory, anti-inflammatory, photoprotective and antimicrobial capacities of the extracts obtained from the eight marine endophytic fungi associated with *Halopteris scoparia*. Dark grey and grey squares illustrate strong and weak bioactive potencies, respectively, and white squares, no biological activity detected.

Concerning the bioactive potential of the crude extracts obtained from these eight endophytic fungi associated with *H*. *scoparia*, the results revealed that all tested extracts exhibited biological activities (Fig 9).

The extract obtained from the fungus *Fusarium incarnatum-equiseti* complex presented the lowest dermocosmetic potential amongst all the tested extracts, considering that it demonstrated weak antioxidant and photoprotective activities, inhibited slightly the hyaluronidase function and *M*. *furfur* growth and, curiously, enhanced the performance of elastase and tyrosinase. Moreover, the extract did not reveal anti-inflammatory activity or interfered on collagenase activity and on *C*. *acnes* and *S*. *epidermidis* growths. This last result contrasts with observations of Zhang et al. [88], who reported an antimicrobial capacity of the compounds produced by an endophytic marine *F*. *oxysporum* against *S*. *epidermis* growth. Despite the irrelevant bioactivities exhibited by the extract from *Fusarium incarnatum-equiseti* complex in this study, additional screening analyses should be carried out with this extract, given the noteworthy results of the few bioprospecting studies already performed with marine strains of *Fusarium* species and the significant number of compounds produced by *Fusarium* species that currently represent lead compounds in therapies against diverse disorders [89]. Another extract apparently with less cosmeceutical potential was the one from *P*. *sizovae*, since it displayed weak antioxidant and photoprotective properties, and only inhibited slightly *M*. *furfur* growth and hyaluronidase and collagenase activities. Nevertheless these results, additional screening tests should also be performed with this extract, considering the fact that this study represents the first screening of the metabolites produced by a marine strain of *P*. *sizovae* and that various *Penicillium* species recovered from marine environments have been demonstrating an ability to secrete compounds with a full range of bioactivities [85]. Corroborating this idea, the extract of *Penicillium* sect. *Exilicaulis* revealed to be one of the most interesting extracts evaluated in this study to be further exploited by the cosmetic industry, since it exhibited mild antioxidant and photoprotective abilities, a slight inhibition of *M*. *furfur* growth, relevant anti-inflammatory properties, and weak and strong inhibitory effects against elastase, and hyaluronidase and collagenase activities, respectively. The fact that two *Penicillium* species isolated from the same host and submitted to the same fermentation conditions biosynthesised secondary metabolites with considerable differences in their biological activities, points out for the existence of different metabolic strategies within this genus.

Similarly, isolates 13 and 20 of *A*. *chevalieri* demonstrated to be producers of very promising extracts for dermocosmetic industry. Specifically, both extracts revealed weak inhibitory effects on hyaluronidase and *S*. *epidermidis* and *M*. *furfur* growths, high inhibitory effect on collagenase activity and *C*. *acnes* growth, and powerful antioxidant capacities. The fact that these extracts did not have any effect on tyrosinase function suggests that the compounds responsible for the antioxidant activities are not interfering in the oxidation of L-DOPA to dopaquinone carried out by this enzyme that represents the second step of melanin biosynthesis [90]. A few differences were though detected between extracts: the extract of isolate 13 showed weak inhibitory potential against elastase activity and photoprotective ability; and the extract of isolate 20, a high capacity to inhibit the collagenase performance and no photoprotection ability. Nevertheless the high and increasing number of bioprospecting studies on marine strains of *Aspergillus* species that are continuously describing new compounds excreted by these species and/or biological activities [86, 87], only a few of them focused on *A*. *chevalieri*. Concretely, Heydari et al. [76] also reported strong antioxidant and antimicrobial abilities against *S*. *epidermidis* and other Gram-positive bacteria in the extract of an isolate of *A*. *chevalieri* associated with a coral, and Bovio et al. [75] referred the antimicrobial activities of the compounds produced by a marine strain isolated from a sponge against pathogenic Gram-positive bacteria.

The remaining extracts demonstrated important but more limited biological activities. The extract of *Diaporthe* sp. revealed a low antioxidant ability, interfered slightly on the hyaluronidase activity and *C*. *acnes* growth, inhibited strongly the collagenase and tyrosinase activities, and revealed a significant anti-inflammatory capacity. The ability of marine strains of *Diaporthe* sp. to biosynthesise bioactive compounds has been evaluated in previous studies [91–93], but thus far, none of the extracts was tested or revealed any of the activities screened in this study. The extract of *A*. *arundinis* had the weakest antioxidative capacity amongst all the extracts and a low inhibitory effect against hyaluronidase, but relevant antimicrobial properties against *M*. *furfur* and a significant anti-inflammatory activity. Similar results were obtained by Hong et al. [39] and Heo et al. [16], who evaluated the antioxidant and tyrosinase inhibition capacities of the compounds biosynthesised by *A*. *arundinis* and other *Arthrinium* species isolated from marine environments, and found that metabolites of *A*. *arundinis* did not present those properties in contrast with the metabolites of other *Arthrinium* species. The extracts from the isolates 2 and 89 of *N*. *oryzae* showed low antioxidant activities and strong anti-inflammatory capacities. However, the extract of isolate 89 revealed also a weak inhibitory effect in hyaluronidase performance, and moderate antimicrobial activities against *S*. *epidermis* and *M*. *furfur*. Previous bioprospecting studies on marine strains of *N*. *oryzae* and other unidentified marine species of *Nigrospora* also demonstrated the antimicrobial potential of several compounds produced by these fungi, but all the bioassays were performed against different microorganisms (*Staphylococcus aureus*, *Bacillus subtilis*, *Micrococcus luteus*, *Streptococcus pneumonia*, *Escherichia coli*, *Enterococcus faecalis*, *Aspergillus sydowii* and *Aspergillus versicolor*) [77, 78, 94, 95]. The extract of *S*. *gracilariae* revealed weak antioxidative and photoprotective abilities, showed a high anti-inflammatory ability, and exerted a slight and strong inhibitory effect against tyrosinase, *S*. *epidermidis* and *M*. *furfur*, and collagenase and *C*. *acnes*, respectively. Though the secondary metabolites of the fungus *S*. *gracilariae* were not, apparently, bioprospected before, those produced by unidentified *Stemphylium* species were also demonstrated by Zhou et al. [96] and Hwang et al. [97] to display antibacterial and anti-inflammatory activities.

The comparison of the results obtained in this study with those already published, highlighted the significant lack of knowledge regarding the fungal biodiversity in marine ecosystems and bioactive potential of fungal species and/or wider taxonomic groups, despite the considerable number of inventorying and bioprospecting studies on marine mycota and, particularly, fungi occurring within seaweeds and marine invertebrates. In fact, and even though most of the biological activities tested in this study have never been tested before, the search for comparable results revealed that the published information is scarce or scattered, which hampered the attempt to combine the information meaningfully and identify probable metabolic patterns within species and/or genus recovered from marine environments. Nonetheless, the integration of all information reinforces the general hypothesis pointed out by several authors, that different fungal strains of the same species might present different metabolic profiles as a result of an adaption strategy to different environments; these differences are even more accentuated if the diversity, potency and/or specificity of bioactive compounds produced by marine and terrestrial fungal strains are compared [5, 35, 85, 98–101]. This assumption is also corroborated by the finding that the extracts from different isolates of *A*. *chevalieri* and *N*. *oryzae*, which were submitted to the same fermentation conditions but recovered from different sampling areas and time periods, exhibited some significant differences in the biological activities.

The increase of knowledge about the ecological niches of fungal species is thus fundamental for a better understanding of the metabolism of each species and the environmental cues that trigger the production of different compounds, and consequently, for an optimisation of the screening methodologies, whether intended to obtain new compounds or maximise the production of particular bioactive compounds. This knowledge depends, in its turn, on a continuous integration of information provided by biodiversity inventories, metabolomic analyses and bioprospecting studies.

Another major finding of this study that should be emphasised is the number and diversity of biological activities with dermocosmetic relevance exhibited by most of the extracts. These results also seem to corroborate the idea that marine endophytic fungal strains associated with seaweeds are prolific producers of metabolites with a wide range of biological properties. Therefore, and as the main purpose of this study was to provide a preliminary screening of the crude extracts, further studies should be performed in order to isolate and identify the compounds responsible for each biological activity of the most promising extracts. Moreover, and given that most of the endophytic fungi reported herein are poorly or have never been bioprospected before, it is of utmost importance to screen other possible biological activities, as well as to evaluate the influence of fermentation conditions in the production of secondary metabolites.

The evaluation of the UV–visible absorption profiles revealed the ability of the extracts obtained from both isolates of *A*. *chevalieri*, *Fusarium incarnatum-equiseti* complex, *Penicillium* sect. *Exilicaulis* and *P*. *sizovae* to absorb in UV-A, UV-B and UV-C regions (Fig 7). These results combined with the slight photoprotective activities exhibited by these extracts (Fig 5), highlighted the potential of extracts to be incorporated into new topical formulations containing natural ingredients with UV-A and UV-B photoprotective effects. In addition, and as reported by Blachowicz et al. [102], the capacity of some fungal species, e.g. *Aspergillus fumigatus* strains, to produce small molecules with UV-C protective potential may contribute to novel safety measures for astronauts, since protection from elevated levels of radiation is of paramount importance for future human outer space explorations.

The fact that both isolates of *A*. *chevalieri* produced compounds with slightly different UV–visible absorption profiles reinforces the influence of environmental conditions in the production of secondary metabolites. Effectively, the extract of isolate 13, which was recovered earlier (June) at Gambôa, exhibited additional absorption peaks in the visible region (400–460 nm) of the spectrum, probably due to the presence of carotenoids, a group of metabolites biosynthesised by several fungi, including *Aspergillus* spp. [103, 104].

The HRMS/MS analysis performed with the extract from isolate 13 enabled the identification of neoechinulin A and echinulin, two indole diketopiperazine alkaloids already reported in extrolites produced by this species [75] and by all *Aspergillus* species included in section *Aspergillus*, regardless the marine or terrestrial origin [105].

Neoechinulin A is one of the most investigated echinulin-related compounds given the wide range of biological activities ascribed to it in previous studies [105, 106]. Kim et al. [107] isolated this compound from the secondary metabolites biosynthesised by a marine *Aspergillus* sp. also included in section *Aspergillus*, and demonstrated that neoechinulin A displayed a strong anti-inflammatory activity in LPS-stimulated RAW264.7 macrophages by suppressing the production of pro-inflammatory mediators and cytokines (nitric oxide, prostaglandin E2, tumor necrosis factor-α, and interleukin-1β). The same effect was observed by Dewapriya et al. [108] with the neoechinulin A isolated from a marine *Microsporum* sp., but in Aβ42-activated BV-2 microglia cells. Li et al. [109] also isolated this metabolite from the culture broth of a marine *Aspergillus* sp., and reported its antioxidant and UV-A protective abilities. Moreover, Maruyama et al. [110], Kimoto et al. [111], Kajimura et al. [112], Aoki et al. [113] and Kamisuki et al. [114] revealed the antiapoptotic and cytoprotective activities of neoechinulin A in neuron-like PC12 cells, against peroxynitrite generated from 3-(4-morpholinyl) sydnonimine hydrochloride (SIN-1) and 1-methyl-4-phenylpyridinium (MPP+). The study carried out by Kimoto et al. [111] also suggested that the antioxidant and anti-nitration activities exhibited by neoechinulin A occurred through an independent mechanism of action. Additionally, Meng et al. [115] referred the moderate brine shrimp lethality activities of both neoechinulin A and echinulin isolated from the secondary metabolites produced by marine mangrove *A*. *ruber* (section *Aspergillus*). Chen et al. [116] and Kamauchi et al. [117] also recovered echinulin from the metabolites produced by a marine *A*. *ruber*, and demonstrated that this extract had a weak antiviral ability and a strong inhibitory activity against melanin synthesis, respectively.

Based on these reports, and though the fact that the bioactivities displayed by the *A*. *chevalieri* extract might result from the combined synergistic or antagonistic effects of all compounds, it is highly likely that the presence of neoechinulin A could explain the strong antioxidant activity of the extract, as well as its photoprotective capacity against UV radiation.

## Conclusions

This study contributed for a better understanding of the diversity and ecology of marine endophytic fungi in general, and of the fungal community inhabiting the brown seaweed *H*. *scoparia*, frequent in the Portuguese west coast, in particular. Specifically, and more importantly, most of the fungal species identified in this study were, for the first time, reported in Portugal, recovered from this seaweed, and bioprospected for bioactive compounds. Moreover, the results revealed that the majority of marine endophytic fungi associated with *H*. *scoparia* were producers of secondary metabolites with promising dermocosmetic potential application. The crude extracts from *A*. *chevalieri* demonstrated to be the most interesting ones for being further and deeply explored by dermocosmetic industry, given the diversity and potency of the biological activities displayed by extracts from both isolates. The presence of neoechinulin A in the extract from isolate 13 of *A*. *chevalieri* could explain the high antioxidant and UV photoprotective capacities exhibited by the extract.

These noteworthy results emphasised the importance of conducting more screening analyses with the extracts from these marine endophytic fungal strains for other potential bioactivities.

This study also pointed out the relevance of investing more efforts in understanding the ecology of halo/osmotolerant fungi, once it will provide relevant clues to infer about the metabolic profile of each species and the factors that might trigger the production of particular compounds. Thus, further inventorying and bioprospecting studies should be carried out in marine ecosystems focusing on different hosts/substrates, habitats and/or geographic/climatic regions.

## Supporting information

S1 Fig Maximum likelihood phylogenetic trees generated from sequences of 1A and BLAST best hits. (PDF)

S2 Fig Evaluation of non-toxic concentrations of fungal extracts to 3T3 cells. (PDF)

S1 Table Means and standard errors of the parameters evaluated in fungal extracts and presented in tables and figures of manuscript, and number of replicates used in each screening approach. (XLSX)

## References

[1] Ebel R. Natural products from marine-derived fungi. In: Jones EBG, Pang K-L, editors. Marine Fungi and Fungal-like Organisms. Berlin, Germany: De Gruyter; 2012. pp. 411–440. 10.1515/9783110264067.411

[2] Carroll AR, Copp BR, Davis RA, Keyzers RA, Prinsep MR. Marine natural products. Natural Product Reports. Royal Society of Chemistry; 2019. pp. 122–173. 10.1039/c8np00092a 30663727

[3] Brakhage AA, Schroeckh V. Fungal secondary metabolites—Strategies to activate silent gene clusters. Fungal Genet Biol. 2011;48: 15–22. 10.1016/j.fgb.2010.04.004 20433937

[4] Imhoff JF. Natural products from marine fungi—Still an underrepresented resource. Mar Drugs. 2016;14: 19. 10.3390/md14010019 26784209 PMC4728516

[5] Reich M, Labes A. How to boost marine fungal research: A first step towards a multidisciplinary approach by combining molecular fungal ecology and natural products chemistry. Mar Genomics. 2017;36: 57–75. 10.1016/j.margen.2017.09.007 29031541

[6] Haefner B. Drugs from the deep: Marine natural products as drug candidates. Drug Discov Today. 2003;8: 536–544. 10.1016/s1359-6446(03)02713-2 12821301

[7] Agrawal S, Adholeya A, Barrow CJ, Deshmukh SK. Marine fungi: An untapped bioresource for future cosmeceuticals. Phytochemistry Letters. Elsevier Ltd; 2018. pp. 15–20. 10.1016/j.phytol.2017.11.003

[8] Martins A, Vieira H, Gaspar H, Santos S. Marketed marine natural products in the pharmaceutical and cosmeceutical industries: Tips for success. Mar Drugs. 2014;12: 1066–1101. 10.3390/md12021066 24549205 PMC3944531

[9] Jenkins G. Molecular mechanisms of skin ageing. Mech Ageing Dev. 2002;123: 801–810. Available: www.elsevier.com/locate/mechagedev 10.1016/s0047-6374(01)00425-0 11869737

[10] Baumann L. Skin ageing and its treatment. J Pathol. 2007;211: 241–251. 10.1002/path.2098 17200942

[11] Farage MA, Miller KW, Elsner P, Maibach HI. Intrinsic and extrinsic factors in skin ageing: A review. Int J Cosmet Sci. 2008;30: 87–95. 10.1111/j.1468-2494.2007.00415.x 18377617

[12] Papakonstantinou E, Roth M, Karakiulakis G. Hyaluronic acid: A key molecule in skin aging. Dermatoendocrinol. 2012;4: 253–258. 10.4161/derm.21923 23467280 PMC3583886

[13] Zhang S, Duan E. Fighting against Skin Aging: The Way from Bench to Bedside. Cell Transplantation. SAGE Publications Ltd; 2018. pp. 729–738. 10.1177/0963689717725755 29692196 PMC6047276

[14] Espinosa-Leal CA, Garcia-Lara S. Current methods for the discovery of new active ingredients from natural products for cosmeceutical applications. Planta Med. 2019;85: 535–551. 10.1055/a-0857-6633 30925621

[15] Kageyama H, Waditee-Sirisattha R. Antioxidative, anti-inflammatory, and anti-aging properties of mycosporine-like amino acids: Molecular and cellular mechanisms in the protection of skin-aging. Mar Drugs. 2019;17: 222. 10.3390/md17040222 31013795 PMC6521297

[16] Heo YM, Kim K, Ryu SM, Kwon SL, Park MY, Kang JE, et al. Diversity and ecology of marine algicolous *Arthrinium* species as a source of bioactive natural products. Mar Drugs. 2018;16: 508. 10.3390/md16120508 30558255 PMC6315899

[17] Corinaldesi C, Barone G, Marcellini F, Dell’Anno A, Danovaro R. Marine microbial-derived molecules and their potential use in cosmeceutical and cosmetic products. Marine Drugs. MDPI AG; 2017. p. 118. 10.3390/md15040118 28417932 PMC5408264

[18] Brown DA. Skin pigmentation enhancers. J Photochem Photobiol B Biol. 2001;63: 148–161. 10.1016/s1011-1344(01)00212-3 11684462

[19] Claudel JP, Auffret N, Leccia MT, Poli F, Corvec S, Dréno B. Staphylococcus epidermidis: A potential new player in the physiopathology of acne? Dermatology. 2019;235: 287–294. 10.1159/000499858 31112983

[20] Gupta A, Batra R, Bluhm R, Boekhout T, Dawson TL. Skin diseases associated with *Malassezia* species. J Am Acad Dermatol. 2004;51: 785–798. 10.1016/j.jaad.2003.12.034 15523360

[21] Theelen B, Cafarchia C, Gaitanis G, Bassukas ID, Boekhout T, Dawson TL. *Malassezia* ecology, pathophysiology, and treatment. Med Mycol. 2018;56: S10–S25. 10.1093/mmy/myx134 29538738

[22] Oren A, Gunde-Cimerman N. Mycosporines and mycosporine-like amino acids: UV protectants or multipurpose secondary metabolites? FEMS Microbiol Lett. 2007;269: 1–10. 10.1111/j.1574-6968.2007.00650.x 17286572

[23] Galasso C, Corinaldesi C, Sansone C. Carotenoids from marine organisms: Biological functions and industrial applications. Antioxidants. 2017;6. 10.3390/antiox6040096 29168774 PMC5745506

[24] Maciel OMC, Tavares RSN, Caluz DRE, Gaspar LR, Debonsi HM. Photoprotective potential of metabolites isolated from algae-associated fungi *Annulohypoxylon stygium*. J Photochem Photobiol B Biol. 2018;178: 316–322. 10.1016/j.jphotobiol.2017.11.018 29175758

[25] Li X, Kim MK, Lee U, Kim S-K, Kang JS, Dae H, et al. Myrothenones A and B, cyclopentenone derivatives with tyrosinase inhibitory activity from the marine-derived fungus *Myrothecium* sp. Chem Pharm Bull. 2005;53: 453–455. 10.1248/cpb.53.453 15802853

[26] Zhang D, Li X, Kang JS, Choi HD, Son BW. A New α-Pyrone derivative, 6-[(E)-Hept-1-enyl]-α-pyrone, with tyrosinase inhibitory activity from a marine isolate of the fungus *Botrytis*. Bull Korean Chem Soc. 2007;28: 887–888.

[27] Wu B, Wu X, Sun M, Li M. Two novel tyrosinase inhibitory sesquiterpenes induced by CuCl2 from a marine-derived fungus Pestalotiopsis sp. Z233. Mar Drugs. 2013;11: 2713–2721. 10.3390/md11082713 23917067 PMC3766860

[28] Tsuchiya T, Yamada K, Minoura K, Miyamoto K, Usami Y, Kobayashi T, et al. Purification and determination of the chemical structure of the tyrosinase inhibitor produced by *Trichoderma viride* strain H1-7 from a marine environment. Biol Pharm Bull. 2008;31: 1618–1620. 10.1248/bpb.31.1618 18670100

[29] Wu B, Wiese J, Labes A, Kramer A, Schmaljohann R, Imhoff JF. Lindgomycin, an unusual antibiotic polyketide from a marine fungus of the Lindgomycetaceae. Mar Drugs. 2015;13: 4617–4632. 10.3390/md13084617 26225984 PMC4556996

[30] Agrawal S, Adholeya A, Barrow CJ, Deshmukh SK. In-vitro evaluation of marine derived fungi against *Cutibacterium acnes*. Anaerobe. 2018;49: 5–13. 10.1016/j.anaerobe.2017.10.010 29100929

[31] Zhang P, Li X, Wang B-G. Secondary metabolites from the marine algal-derived endophytic fungi: Chemical diversity and biological activity. Planta Med. 2016;82: 832–842. 10.1055/s-0042-103496 27220083

[32] Tan RX, Zou WX. Endophytes: A rich source of functional metabolites. Nat Prod Rep. 2001;18: 448–459. 10.1039/b100918o 11548053

[33] Nicoletti R, Salvatore MM, Andolfi A. Secondary metabolites of mangrove-associated strains of Talaromyces. Mar Drugs. 2018;16: 12. 10.3390/md16010012 29316607 PMC5793060

[34] Zuccaro A, Schulz B, Mitchell JI. Molecular detection of ascomycetes associated with *Fucus serratus*. Mycol Res. 2003;107: 1451–1466. 10.1017/s0953756203008657 15000246

[35] Suryanarayanan TS, Venkatachalam A, Thirunavukkarasu N, Ravishankar JP, Doble M, Geetha V. Internal mycobiota of marine macroalgae from the Tamilnadu coast: Distribution, diversity and biotechnological potential. Bot 3. 2010;53: 457–468. 10.1515/BOT.2010.045

[36] Gnavi G, Palma Esposito F, Festa C, Poli A, Tedesco P, Fani R, et al. The antimicrobial potential of algicolous marine fungi for counteracting multidrug-resistant bacteria: phylogenetic diversity and chemical profiling. Res Microbiol. 2016;167: 492–500. 10.1016/j.resmic.2016.04.009 27154031

[37] Vallet M, Strittmatter M, Murúa P, Lacoste S, Dupont J, Hubas C, et al. Chemically-mediated interactions between macroalgae, their fungal endophytes, and protistan pathogens. Front Microbiol. 2018;9: 1–13. 10.3389/fmicb.2018.00001 30627120 PMC6309705

[38] Youssef FS, Ashour ML, Singab ANB, Wink M. A comprehensive review of bioactive peptides from marine fungi and their biological significance. Mar Drugs. 2019;17: 559. 10.3390/md17100559 31569458 PMC6835287

[39] Hong J-H, Jang S, Heo YM, Min M, Lee H, Lee YM, et al. Investigation of marine-derived fungal diversity and their exploitable biological activities. Mar Drugs. 2015;13: 4137–4155. 10.3390/md13074137 26133554 PMC4515608

[40] Singh RP, Kumari P, Reddy CRK. Antimicrobial compounds from seaweeds-associated bacteria and fungi. Appl Microbiol Biotechnol. 2015;99: 1571–1586. 10.1007/s00253-014-6334-y 25549621

[41] Overy DP, Rämä T, Oosterhuis R, Walker AK, Pang K-L. The neglected marine fungi, sensu stricto, and their isolation for natural products’ discovery. Mar Drugs. 2019;17: 42. 10.3390/md17010042 30634599 PMC6356354

[42] Patarra RF, Carreiro AS, Lloveras AA, Abreu MH, Buschmann AH, Neto AI. Effects of light, temperature and stocking density on Halopteris scoparia growth. J Appl Phycol. 2017;29: 405–411. 10.1007/s10811-016-0933-1

[43] Güner A, Nalbantsoy A, Sukatar A, Karabay Yavaşoğlu NÜ. Apoptosis-inducing activities of Halopteris scoparia L. Sauvageau (Brown algae) on cancer cells and its biosafety and antioxidant properties. Cytotechnology. 2019;71: 687–704. 10.1007/s10616-019-00314-5 30969390 PMC6546795

[44] Liu D, Coloe S, Baird R, Pederson J. Rapid Mini-Preparation of Fungal DNA for PCR Nucleic. J Clin Microbiol. 2000;38: 471. 10681211 10.1128/jcm.38.1.471-471.2000 PMC88759

[45] White TJ, Bruns T, Lee S, Taylor J. Amplification and direct sequencing of fungal ribosomal RNA Genes for phylogenetics. In: Innis MA, Gelfand DH, Sninsky JJ, White TJ, editors. PCR Protocols: A guide to Methods and Applications. Academic Press; 1990. pp. 315–322. Available: https://www.researchgate.net/publication/223397588

[46] Nilsson RH, Tedersoo L, Abarenkov K, Ryberg M, Kristiansson E, Hartmann M, et al. Five simple guidelines for establishing basic authenticity and reliability of newly generated fungal ITS sequences. MycoKeys. 2012;4: 37–63. 10.3897/mycokeys.4.3606

[47] Blaalid R, Kumar S, Nilsson RH, Abarenkov K, Kirk PM, Kauserud H. ITS1 versus ITS2 as DNA metabarcodes for fungi. Mol Ecol Resour. 2013;13. 10.1111/1755-0998.12065 23350562

[48] Schoch CL, Seifert KA, Huhndorf S, Robert V, Spouge JL, Levesque CA, et al. Nuclear ribosomal internal transcribed spacer (ITS) region as a universal DNA barcode marker for Fungi. Proc Natl Acad Sci U S A. 2012;109: 6241–6246. 10.1073/pnas.1117018109 22454494 PMC3341068

[49] Samson RA, Visagie CM, Houbraken J, Hong S-B, Hubka V, Klaassen CHW, et al. Phylogeny, identification and nomenclature of the genus Aspergillus. Studies in Mycology. Centraalbureau voor Schimmelculturen; 2014. 10.1016/j.simyco.2014.07.004 25492982 PMC4260807

[50] Woudenberg JHC, Meijer M, Houbraken J, Samson RA. Scopulariopsis and scopulariopsis-like species from indoor environments. Stud Mycol. 2017;88: 1–35. 10.1016/j.simyco.2017.03.001 28413236 PMC5384890

[51] Hilário S, Amaral IA, Gonçalves MFM, Lopes A, Santos L, Alves A. *Diaporthe* species associated with twig blight and dieback of Vaccinium corymbosum in Portugal, with description of four new species. Mycologia. 2020;112: 293–308. 10.1080/00275514.2019.1698926 32074022

[52] Visagie CM, Houbraken J, Frisvad JC, Hong S-B, Klaassen CHW, Perrone G, et al. Identification and nomenclature of the genus Penicillium. Studies in Mycology. Centraalbureau voor Schimmelculturen; 2014. 10.1016/j.simyco.2014.09.001 25505353 PMC4261876

[53] Visagie CM, Seifert KA, Houbraken J, Samson RA, Jacobs K. A phylogenetic revision of Penicillium sect. Exilicaulis, including nine new species from fynbos in South Africa. IMA Fungus. 2016;7: 75–117. 10.5598/imafungus.2016.07.01.06 27433442 PMC4941689

[54] Karlsson I, Edel-Hermann V, Gautheron N, Durling MB, Kolseth AK, Steinberg C, et al. Genus-specific primers for study of *Fusarium* communities in field samples. Appl Environ Microbiol. 2016;82: 491–501. 10.1128/AEM.02748-15 26519387 PMC4711133

[55] Yang Q, Fan X-L, Guarnaccia V, Tian C-M. High diversity of Diaporthe species associated with dieback diseases in China, with twelve new species described. MycoKeys. 2018;39: 97–149. 10.3897/mycokeys.39.26914 30271260 PMC6160862

[56] Kumar S, Stecher G, Li M, Knyaz C, Tamura K. MEGA X: Molecular evolutionary genetics analysis across computing platforms. Mol Biol Evol. 2018;35: 1547–1549. 10.1093/molbev/msy096 29722887 PMC5967553

[57] Kjer J, Debbab A, Aly AH, Proksch P. Methods for isolation of marine-derived endophytic fungi and their bioactive secondary products. Nat Protoc. 2010;5: 479–490. 10.1038/nprot.2009.233 20203665

[58] Tarman K, Lindequist U, Wende K, Porzel A, Arnold N, Wessjohann LA. Isolation of a new natural product and cytotoxic and antimicrobial activities of extracts from fungi of Indonesian marine habitats. Mar Drugs. 2011;9: 294–306. 10.3390/md9030294 21556160 PMC3083651

[59] Pinteus S, Silva J, Alves C, Horta A, Fino N, Rodrigues AI, et al. Cytoprotective effect of seaweeds with high antioxidant activity from the Peniche coast (Portugal). Food Chem. 2017;218: 591–599. 10.1016/j.foodchem.2016.09.067 27719954

[60] Silva J, Alves C, Freitas R, Martins A, Pinteus S, Ribeiro J, et al. Antioxidant and neuroprotective potential of the brown seaweed Bifurcaria bifurcata in an in vitro Parkinson’s disease model. Mar Drugs. 2019;17: 85. 10.3390/md17020085 30717087 PMC6410415

[61] Yahaya YA, Don MM. Evaluation of *Trametes lactinea* extracts on the inhibition of hyaluronidase, lipoxygenase and xanthine oxidase activities in Vitro. J Phys Sci. 2012;23: 1–15.

[62] Lee KH, Farida FH, Syahida A, Abas F, Shaari K, Israf DA, et al. Synthesis and biological evaluation of curcumin-like diarylpentanoid analogues for anti-inflammatory, antioxidant and anti-tyrosinase activities. Eur J Med Chem. 2009;44: 3195–3200. 10.1016/j.ejmech.2009.03.020 19359068

[63] Senol FS, Orhan IE, Ozgen U, Renda G, Bulut G, Guven L, et al. Memory-vitalizing effect of twenty-five medicinal and edible plants and their isolated compounds. South African J Bot. 2016;102: 102–109. 10.1016/j.sajb.2015.07.011

[64] Mosmann T. Rapid colorimetric assay for cellular growth and survival: Application to proliferation and cytotoxicity assays. J Immunol Methods. 1983;65: 55–63. 10.1016/0022-1759(83)90303-4 6606682

[65] Yang EJ, Yim EY, Song G, Kim GO, Hyun CG. Inhibition of nitric oxide production in lipopolysaccharide-activated RAW 264.7 macrophages by Jeju plant extracts. Interdiscip Toxicol. 2009;2: 245–249. 10.2478/v10102-009-0022-2 21217861 PMC2984114

[66] Marto J, Neves Â, Gonçalves LM, Pinto P, Almeida C, Simões S. Rice water: A traditional ingredient with anti-aging efficacy. Cosmetics. 2018;5: 1–12. 10.3390/cosmetics5020026

[67] Horta A, Pinteus S, Alves C, Fino N, Silva J, Fernandez S, et al. Antioxidant and antimicrobial potential of the Bifurcaria bifurcata epiphytic bacteria. Mar Drugs. 2014;12: 1676–1689. 10.3390/md12031676 24663118 PMC3967231

[68] Santos L, Alves A, Alves R. Evaluating multi-locus phylogenies for species boundaries determination in the genus Diaporthe. PeerJ. 2017;2017. 10.7717/peerj.3120 28367371 PMC5372842

[69] Tamura K, Nei M. Estimation of the number of nucleotide substitutions in the control region of mitochondrial DNA in humans and chimpanzees. Mol Biol Evol. 1993;10: 512–526. 10.1093/oxfordjournals.molbev.a040023 8336541

[70] Panno L, Bruno M, Voyron S, Anastasi A, Gnavi G, Miserere L, et al. Diversity, ecological role and potential biotechnological applications of marine fungi associated to the seagrass Posidonia oceanica. N Biotechnol. 2013;30: 685–694. 10.1016/j.nbt.2013.01.010 23410985

[71] Wang J, Wei X, Qin X, Lin X, Zhou X, Liao S, et al. Arthpyrones A-C, pyridone alkaloids from a sponge-derived fungus *Arthrinium arundinis* ZSDS1-F3. Org Lett. 2015;17: 656–659. 10.1021/ol503646c 25606827

[72] Garzoli L, Poli A, Prigione V, Gnavi G, Varese GC. Peacock’s tail with a fungal cocktail: first assessment of the mycobiota associated with the brown alga Padina pavonica. Fungal Ecol. 2018;35: 87–97. 10.1016/j.funeco.2018.05.005

[73] Furtado BU, Szymańska S, Hrynkiewicz K. A window into fungal endophytism in Salicornia europaea: deciphering fungal characteristics as plant growth promoting agents. Plant Soil. 2019. 10.1007/s11104-019-04315-3

[74] Wiese J, Ohlendorf B, Blümel M, Schmaljohann R, Imhoff JF. Phylogenetic identification of fungi isolated from the marine sponge Tethya aurantium and identification of their secondary metabolites. Mar Drugs. 2011;9: 561–585. 10.3390/md9040561 21731550 PMC3124973

[75] Bovio E, Garzoli L, Poli A, Luganini A, Villa P, Musumeci R, et al. Marine Fungi from the sponge Grantia compressa: Biodiversity, chemodiversity, and biotechnological potential. Mar Drugs. 2019;17: 220. 10.3390/md17040220 30978942 PMC6520677

[76] Heydari H, Koc A, Simsek D, Gozcelioglu B, Altanlar N, Konuklugil B. Isolation, identification and bioactivity screening of Turkish marine-derived fungi. Farmacia. 2019;67: 780–788. 10.31925/farmacia.2019.5.5

[77] Dong J-J, Bao J, Zhang X-Y, Xu X-Y, Nong X-H, Qi S-H. Alkaloids and citrinins from marine-derived fungus *Nigrospora oryzae* SCSGAF 0111. Tetrahedron Lett. 2014;55: 2749–2753. 10.1016/j.tetlet.2014.03.060

[78] Ding L-J, Yuan W, Liao X-J, Han B-N, Wang S-P, Li Z-Y, et al. Oryzamides A-E, cyclodepsipeptides from the sponge-derived fungus *Nigrospora oryzae* PF18. J Nat Prod. 2016;79: 2045–2052. 10.1021/acs.jnatprod.6b00349 27489998

[79] Abdel-Wahab MA, Bahkali AHA. Taxonomy of filamentous anamorphic marine fungi: morphology and molecular evidence. In: Jones EBG, Pang K-L, editors. Marine Fungi and Fungal-like Organisms. Berlin, Germany: De Gruyter; 2012. pp. 65–90. 10.1515/9783110264067.65

[80] Woudenberg JHC, Hanse B, van Leeuwen GCM, Groenewald JZ, Crous PW. Stemphylium revisited. Stud Mycol. 2017;87: 77–103. 10.1016/j.simyco.2017.06.001 28663603 PMC5480992

[81] Bovio E, Gnavi G, Prigione V, Spina F, Denaro R, Yakimov M, et al. The culturable mycobiota of a Mediterranean marine site after an oil spill: Isolation, identification and potential application in bioremediation. Sci Total Environ. 2017;576: 310–318. 10.1016/j.scitotenv.2016.10.064 27788446

[82] Butinar L, Frisvad JC, Gunde-Cimerman N. Hypersaline waters-a potential source of foodborne toxigenic aspergilli and penicillia. FEMS Microbiol Ecol. 2011;77: 186–199. 10.1111/j.1574-6941.2011.01108.x 21477006

[83] Zuccaro A, Schoch CL, Spatafora JW, Kohlmeyer J, Draeger S, Mitchell JI. Detection and identification of fungi intimately associated with the brown seaweed *Fucus serratus*. Appl Environ Microbiol. 2008;74: 931–941. 10.1128/AEM.01158-07 18083854 PMC2258598

[84] Venkatachalam A, Govinda Rajulu MB, Thirunavukkarasu N, Suryanarayanan TS. Endophytic fungi of marine algae and seagrasses: A novel source of chitin modifying enzymes. Mycosphere. 2015;6: 345–355. 10.5943/MYCOSPHERE/6/3/10

[85] Nicoletti R, Trincone A. Bioactive compounds produced by strains of Penicillium and Talaromyces of marine origin. Mar Drugs. 2016;14: 37. 10.3390/md14020037 26901206 PMC4771990

[86] Lee YM, Kim MJ, Li H, Zhang P, Bao B, Lee KJ, et al. Marine-derived *Aspergillus* species as a source of bioactive secondary metabolites. Mar Biotechnol. 2013;15: 499–519. 10.1007/s10126-013-9506-3 23709045

[87] Xu L, Meng W, Cao C, Wang J, Shan W, Wang Q. Antibacterial and antifungal compounds from marine fungi. Mar Drugs. 2015;13: 3479–3513. 10.3390/md13063479 26042616 PMC4483641

[88] Zhang Y, Mu J, Feng Y, Kang Y, Zhang J, Gu PJ, et al. Broad-spectrum antimicrobial epiphytic and endophytic fungi from marine organisms: Isolation, bioassay and taxonomy. Mar Drugs. 2009;7: 97–112. 10.3390/md7020097 19597575 PMC2707037

[89] Toghueo RMK. Bioprospecting endophytic fungi from Fusarium genus as sources of bioactive metabolites. Mycology. 2020;11: 1–21. 10.1080/21501203.2019.1645053 32128278 PMC7033707

[90] Uchida R, Ishikawa S, Tomoda H. Inhibition of tyrosinase activity and melanine pigmentation by 2-hydroxytyrosol. Acta Pharm Sin B. 2014;4: 141–145. 10.1016/j.apsb.2013.12.008 26579376 PMC4590298

[91] Cui H, Yu J, Chen S, Ding M, Huang X, Yuan J, et al. Alkaloids from the mangrove endophytic fungus *Diaporthe phaseolorum* SKS019. Bioorganic Med Chem Lett. 2017;27: 803–807. 10.1016/j.bmcl.2017.01.029 28119026

[92] Luo X, Yang J, Chen F, Lin X, Chen C, Zhou X, et al. Structurally diverse polyketides from the mangrove-derived fungus Diaporthe sp. SCSIO 41011 with their anti-influenza A virus activities. Front Chem. 2018;6. 10.3389/fchem.2018.00006 30050898 PMC6052247

[93] Lin X, Huang Y, Fang M, Wang J, Zheng Z, Su W. Cytotoxic and antimicrobial metabolites from marine lignicolous fungi, *Diaporthe* sp. FEMS Microbiol Lett. 2005;251: 53–58. 10.1016/j.femsle.2005.07.025 16102912

[94] Arumugam GK, Srinivasan SK, Joshi G, Gopal D, Ramalingam K. Production and characterization of bioactive metabolites from piezotolerant deep sea fungus *Nigrospora* sp. in submerged fermentation. J Appl Microbiol. 2014;118: 99–111. 10.1111/jam.12693 25393321

[95] Trisuwan K, Rukachaisirikul V, Sukpondma Y, Preedanon S, Phongpaichit S, Sakayaroj J. Pyrone derivatives from the marine-derived fungus *Nigrospora* sp. PSU-F18. Phytochemistry. 2009;70: 554–557. 10.1016/j.phytochem.2009.01.008 19237178

[96] Zhou X-M, Zheng C-J, Chen G-Y, Song X-P, Han C-R, Li G-N, et al. Bioactive anthraquinone derivatives from the mangrove-derived fungus *Stemphylium* sp. 33231. J Nat Prod. 2014;77: 2021–2028. 10.1021/np500340y 25136754

[97] Hwang J-Y, Park SC, Byun WS, Oh D-C, Lee SK, Oh K-B, et al. Bioactive bianthraquinones and meroterpenoids from a marine-derived Stemphylium sp. fungus. Mar Drugs. 2020; 1–19. 10.3390/md19010001 32825785 PMC7551059

[98] Masuma R, Yamaguchi Y, Noumi M, Omura S, Namikoshi M. Effect of sea water concentration on hyphal growth and antimicrobial metabolite production in marine fungi. Mycoscience. 2001;42: 455–459.

[99] Damare S, Raghukumar C, Raghukumar S. Fungi in deep-sea sediments of the Central Indian Basin. Deep Res Part I Oceanogr Res Pap. 2006;53: 14–27. 10.1016/j.dsr.2005.09.005

[100] Smetanina OF, Kalinovskii AI, Khudyakova Y V, Slinkina NN, Pivkin M V, Kuznetsova TA. Metabolites from the marine fungus *Eurotium repens*. Chem Nat Compd. 2007;43: 395–398.

[101] Huang J, Lu C, Qian X, Huang Y, Zheng Z, Shen Y. Effect of salinity on the growth, biological activity and secondary metabolites of some marine fungi. Acta Oceanol Sin. 2011;30: 118–123. 10.1007/s13131-011-0126-3

[102] Blachowicz A, Raffa N, Bok JW, Choera T, Knox B, Lim FY, et al. Contributions of spore secondary metabolites to UV-C protection and virulence vary in different Aspergillus fumigatus strains. 2020;11: 1–12. 10.1128/mBio.03415-19 PMC7029147 32071276

[103] Avalos J, Carmen Limón M. Biological roles of fungal carotenoids. Curr Genet. 2015;61: 309–324. 10.1007/s00294-014-0454-x 25284291

[104] van Eijk GW, Mummery RS, Roeymans HJ, Valadon LRG. A comparative study of carotenoids of *Aschersonia aleyroides* and *Aspergillus giganteus*. Antonie Van Leeuwenhoek. 1979;45: 417–422. 10.1007/BF00443280 554534

[105] Chen AJ, Hubka V, Frisvad JC, Visagie CM, Houbraken J, Meijer M, et al. Polyphasic taxonomy of Aspergillus section Aspergillus (formerly Eurotium), and its occurrence in indoor environments and food. Studies in Mycology. CBS-KNAW Fungal Biodiversity Centre; 2017. 10.1016/j.simyco.2017.07.001 28860671 PMC5573881

[106] Wang X, Li Y, Zhang X, Lai D, Zhou L. Structural diversity and biological activities of the cyclodipeptides from Fungi. Molecules. 2017;22. 10.3390/molecules22122026 29168781 PMC6149763

[107] Kim KS, Cui X, Lee DS, Sohn JH, Yim JH, Kim YC, et al. Anti-inflammatory effect of neoechinulin A from the marine fungus Eurotium sp. SF-5989 through the suppression of NF-?B and p38 MAPK pathways in lipopolysaccharide-stimulated RAW264.7 macrophages. Molecules. 2013;18: 13245–13259. 10.3390/molecules181113245 24165583 PMC6270177

[108] Dewapriya P, Li YX, Himaya SWA, Pangestuti R, Kim SK. Neoechinulin A suppresses amyloid-β oligomer-induced microglia activation and thereby protects PC-12 cells from inflammation-mediated toxicity. Neurotoxicology. 2013;35: 30–40. 10.1016/j.neuro.2012.12.004 23261590

[109] Li Y, Li X, Kim S-K, Kang JS, Choi HD, Rho JR, et al. Golmaenone, a new diketopiperazine alkaloid from the marine-derived fungus Aspergillus sp. Chem Pharm Bullettin. 2004;52: 375–376. 10.1248/cpb.52.375 14993767

[110] Maruyama K, Ohuchi T, Yoshida K, Shibata Y, Sugawara F, Arai T. Protective properties of neoechinulin A against SIN-1-induced neuronal cell death. J Biochem. 2004;136: 81–87. 10.1093/jb/mvh103 15269243

[111] Kimoto K, Aoki T, Shibata Y, Kamisuki S, Sugawara F, Kuramochi K, et al. Structure-activity relationships of neoechinulin A analogues with cytoprotection against peroxynitrite-induced PC12 cell death. J Antibiot (Tokyo). 2007;60: 614–621. 10.1038/ja.2007.79 17965477

[112] Kajimura Y, Aoki T, Kuramochi K, Kobayashi S, Sugawara F, Watanabe N, et al. Neoechinulin a protects PC12 cells against MPP+-induced cytotoxicity. J Antibiot (Tokyo). 2008;61: 330–333. 10.1038/ja.2008.48 18654001

[113] Aoki T, Ohnishi K, Kimoto M, Fujieda S, Kuramochi K, Takeuchi T, et al. Synthesis and neuroprotective action of optically pure neoechinulin A and its analogs. Pharmaceuticals. 2010;3: 1063–1069. 10.3390/ph3041063 27713287 PMC4034020

[114] Kamisuki S, Himeno N, Tsurukawa Y, Kusayanagi T, Takeno M, Kamakura T, et al. Identification of proteins that bind to the neuroprotective agent neoechinulin A. Biosci Biotechnol Biochem. 2018;82: 442–448. 10.1080/09168451.2018.1433018 29447077

[115] Meng LH, Du FY, Li XM, Pedpradab P, Xu GM, Wang BG. Rubrumazines A-C, indolediketopiperazines of the isoechinulin class from *Eurotium rubrum* MA-150, a fungus obtained from marine mangrove-derived rhizospheric soil. J Nat Prod. 2015;78: 909–913. 10.1021/np5007839 25730346

[116] Chen X, Si L, Liu D, Proksch P, Zhang L, Zhou D, et al. Neoechinulin B and its analogues as potential entry inhibitors of influenza viruses, targeting viral hemagglutinin. Eur J Med Chem. 2015;93: 182–195. 10.1016/j.ejmech.2015.02.006 25681711

[117] Kamauchi H, Kinoshita K, Sugita T, Koyama K. Conditional changes enhanced production of bioactive metabolites of marine derived fungus *Eurotium rubrum*. Bioorganic Med Chem Lett. 2016;26: 4911–4914. 10.1016/j.bmcl.2016.09.017 27641468

