# Supplementary material for: Marine endophytic fungi associated with Halopteris scoparia (Linnaeus) Sauvageau as producers of bioactive secondary metabolites with potential dermocosmetic application
Source: PLoS One. 2021 May 13;16(5):e0250954. doi: 10.1371/journal.pone.0250954 (PMC8118457; doi:10.1371/journal.pone.0250954)
Supplement: S1 Fig — (PDF) [file pone.0250954.s001.pdf]

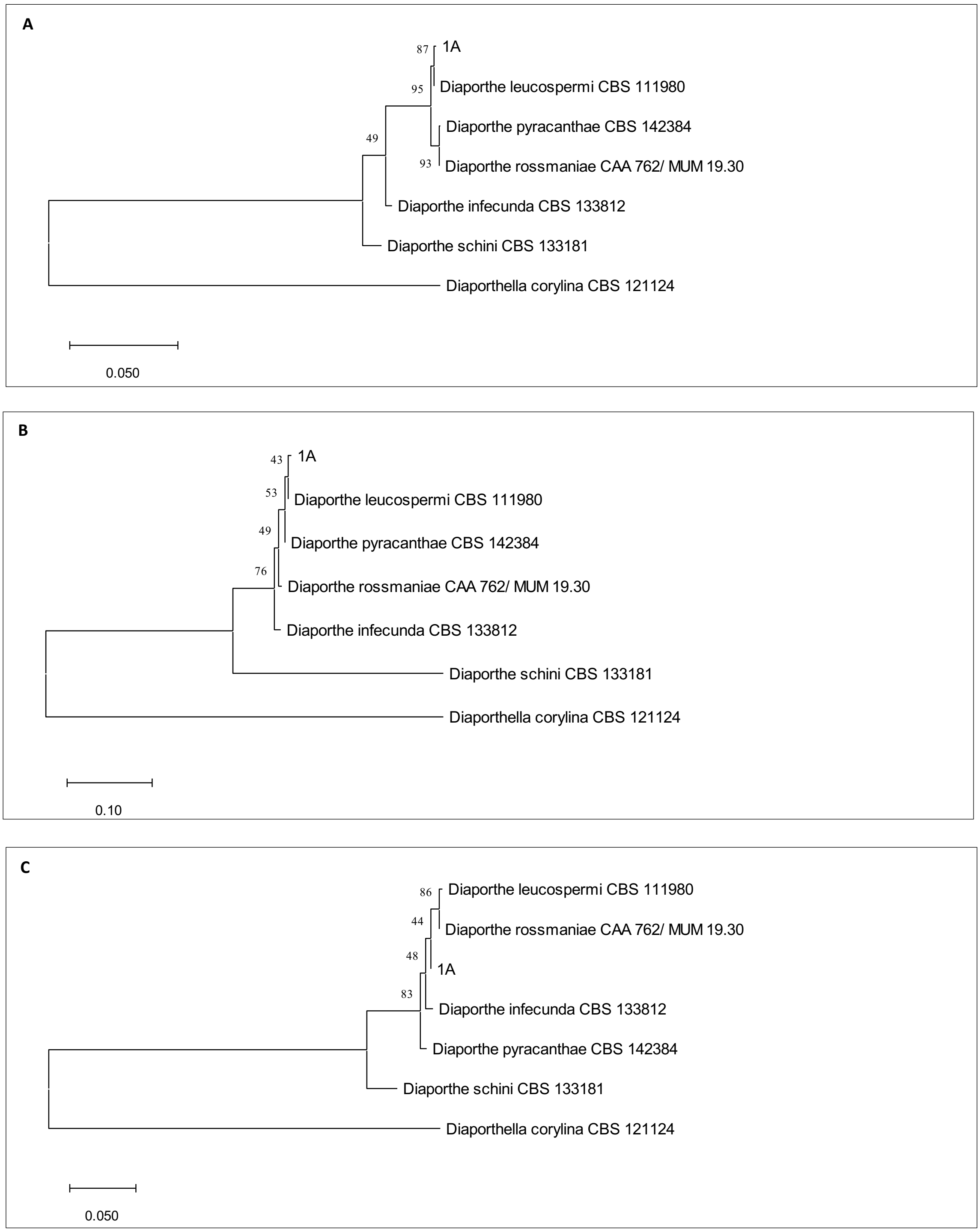
A
87
1A
95
Diaporthe leucospermi CBS 111980
Diaporthe pyracanthae CBS 142384
49
93
Diaporthe rossmaniae CAA 762/ MUM 19.30
Diaporthe infecunda CBS 133812
Diaporthe schini CBS 133181
Diaporthella corylina CBS 121124
0.050
B
43
1A
53
Diaporthe leucospermi CBS 111980
49
Diaporthe pyracanthae CBS 142384
76
Diaporthe rossmaniae CAA 762/ MUM 19.30
Diaporthe infecunda CBS 133812
Diaporthe schini CBS 133181
Diaporthella corylina CBS 121124
0.10
C
86
Diaporthe leucospermi CBS 111980
44
Diaporthe rossmaniae CAA 762/ MUM 19.30
48
1A
83
Diaporthe infecunda CBS 133812
Diaporthe pyracanthae CBS 142384
Diaporthe schini CBS 133181
Diaporthella corylina CBS 121124
0.050

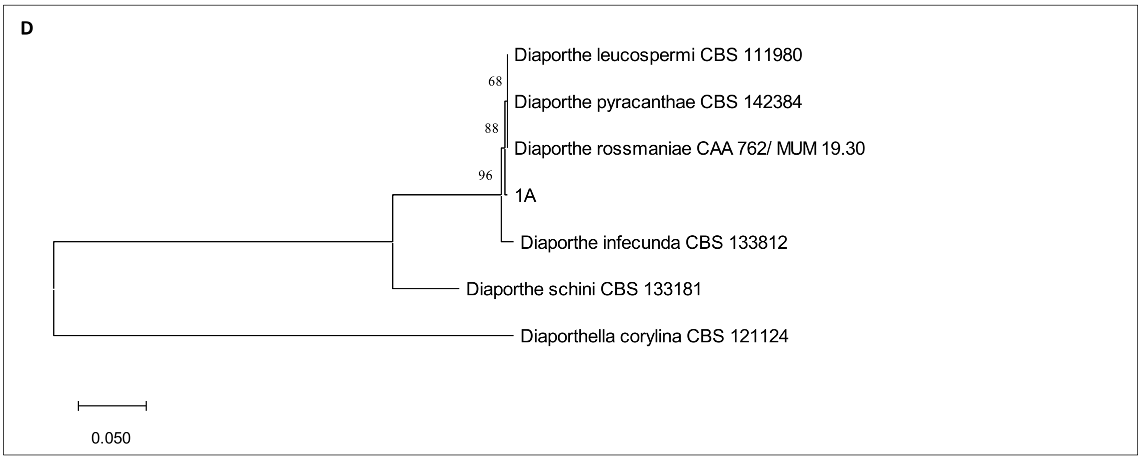

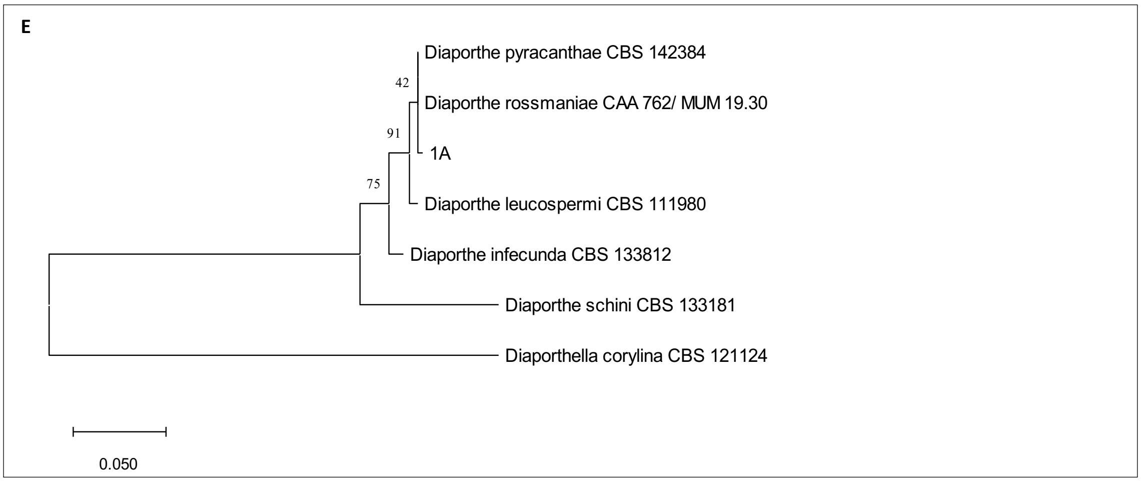

S1 Fig. Maximum likelihood phylogenetic trees inferred from the datasets of ITS (A), tef1-α (B), β-tubulin (C), cal (D) and his3 (E) sequences of isolate 1A and BLAST best hits. *Diaporthella corylina* was used as outgroup. ML analysis was based on the Tamura-Nei model and the tree was obtained from an initial bio-neighbour-joining tree automatically generated by the software, followed by a heuristic search using the nearest-neighbor interchange algorithm. A discrete gamma distribution was used to model evolutionary rate differences among sites. The percentage of trees in which the associated taxa clustered together is shown next to the branches.
