# Supplementary material for: Marine endophytic fungi associated with Halopteris scoparia (Linnaeus) Sauvageau as producers of bioactive secondary metabolites with potential dermocosmetic application
Source: PLoS One. 2021 May 13;16(5):e0250954. doi: 10.1371/journal.pone.0250954 (PMC8118457; doi:10.1371/journal.pone.0250954)
Supplement: S2 Fig — (PDF) [file pone.0250954.s002.pdf]

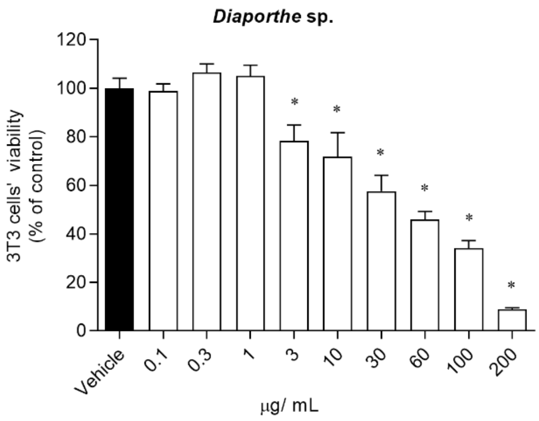
Diaporthe sp.
3T3 cells' viability (% of control)
0
20
40
60
80
100
120
Vehicle
0.1
0.3
1
3
10
30
60
100
200
μg/ mL
*

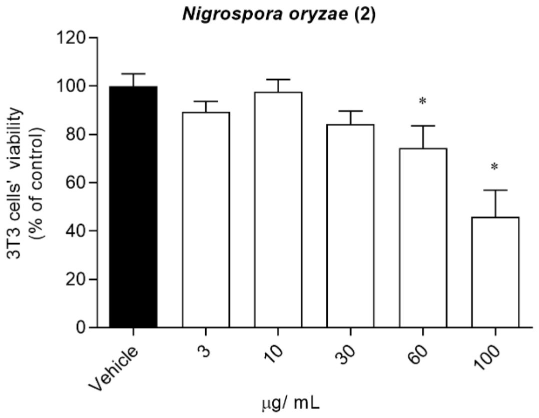
Nigrospora oryzae (2)
3T3 cells' viability (% of control)
0
20
40
60
80
100
120
Vehicle
3
10
30
60
100
μg/ mL
*

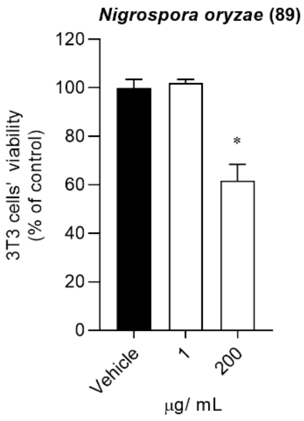
Nigrospora oryzae (89)
3T3 cells' viability (% of control)
0
20
40
60
80
100
120
Vehicle
1
200
μg/ mL
*

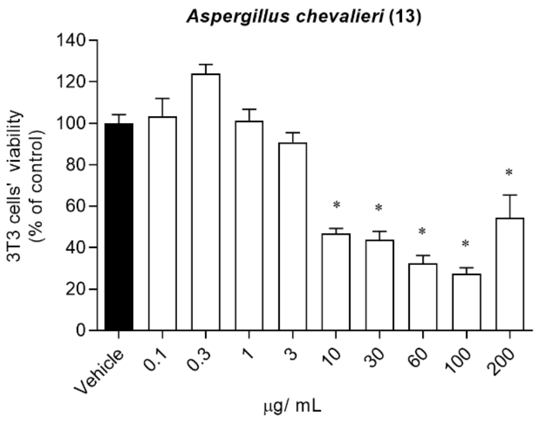
Aspergillus chevalieri (13)
3T3 cells' viability (% of control)
0
20
40
60
80
100
120
140
Vehicle
0.1
0.3
1
3
10
30
60
100
200
μg/ mL
*

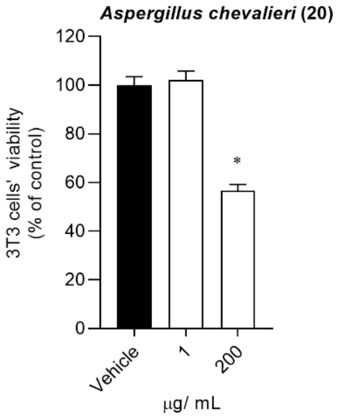
Aspergillus chevalieri (20)
3T3 cells' viability (% of control)
0
20
40
60
80
100
120
Vehicle
1
200
μg/ mL
*

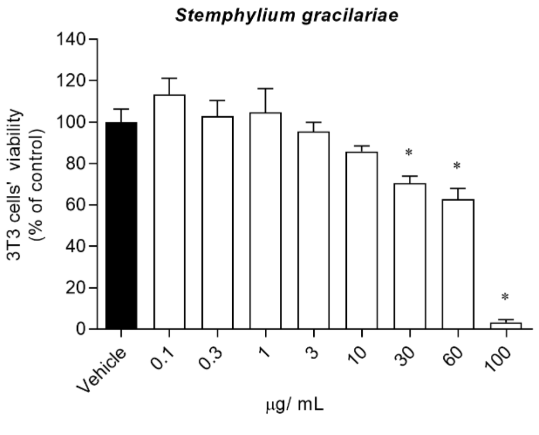
Stemphylium gracilariae
3T3 cells' viability (% of control)
0
20
40
60
80
100
120
140
Vehicle
0.1
0.3
1
3
10
30
60
100
μg/ mL
*

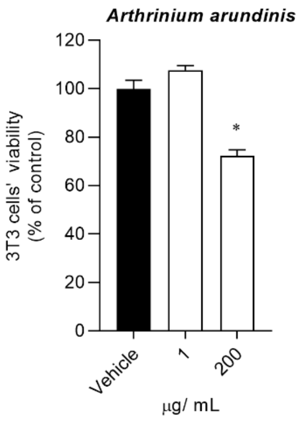

Arthrinium arundinis
3T3 cells' viability (% of control)
0
20
40
60
80
100
120
Vehicle
1
200
μg/ mL

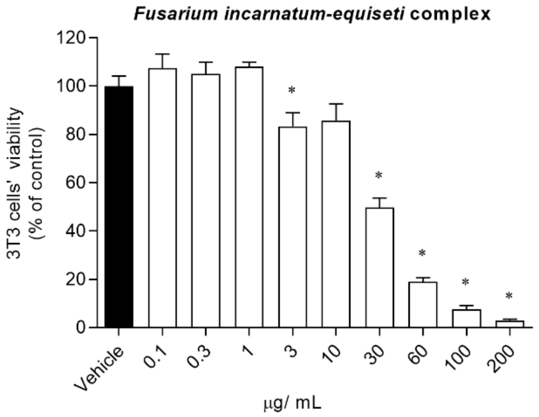

Fusarium incarnatum-equiseti complex
3T3 cells' viability (% of control)
0
20
40
60
80
100
120
Vehicle
0.1
0.3
1
3
10
30
60
100
200
μg/ mL

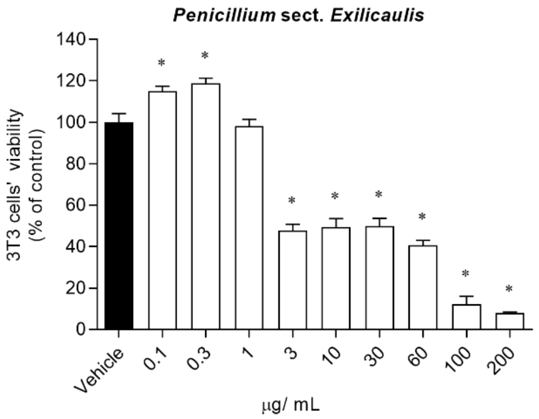

Penicillium sect. *Exilicaulis*
3T3 cells' viability (% of control)
0
20
40
60
80
100
120
140
Vehicle
0.1
0.3
1
3
10
30
60
100
200
μg/ mL

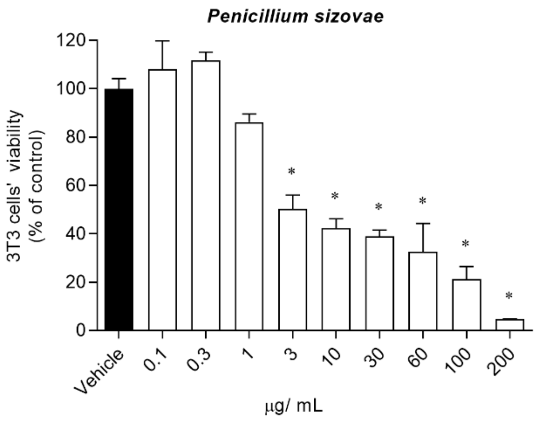

S2 Fig. 3T3 cells' viability following 24 h of exposure to the crude extracts obtained from endophytic fungi associated with *Halopteris scoparia* (0.1-200 µg/ mL) and expressed as % of the control. The values correspond to mean ± SEM. * represents significant differences (One-way ANOVA, Dunnett's test; $p< 0.05$) when compared with the vehicle.
